# An Annotated Checklist of Symbiotic Copepods of Mollusks in the Global Oceans: A Review of Diversity, Hosts and Geographical Distributions

**DOI:** 10.3390/ani16020212

**Published:** 2026-01-10

**Authors:** Jing Sun, Huidong Ju, Xin Du, Congmei Xu, Muhammad Saleem Chang, Ziteng Liu, Xiaobing Li

**Affiliations:** 1 Ocean College, Hebei Agricultural University, Qinhuangdao 066000, China; 2 Hebei Key Laboratory of Nutritional Regulation and Disease Control for Aquaculture, Qinhuangdao 066000, China; 3 Department of Agriculture and Food Science, Shijiazhuang University, Shijiazhuang 050037, China; 4 College of Advanced Agriculture and Life Sciences, Weifang University, Weifang 261061, China; 5 Department of Science and Technical Education, Faculty of Education, University of Sindh, Jamshoro 76080, Pakistan; 6 College of Chemical Engineering, Shijiazhuang University, Shijiazhuang 050037, China

**Keywords:** copepods, mollusks, diversity, hosts, geographical distributions

## Abstract

Symbiotic copepods have a wide host group, including not only invertebrates but also vertebrates, with variable symbiotic sites and morphological characteristics. Even though symbiotic copepods exhibit remarkable diversity, our knowledge of them is still very limited, causing significant lacunae in our understanding of their taxonomic characteristics, host associations, and geographical distributions. To fill these knowledge gaps, we have compiled a comprehensive list of symbiotic copepods and their molluscan hosts in the global oceans based on an extensive literature review. The inventory provides a comprehensive synthesis of the diversity, hosts, and geographical distributions of the symbiotic copepods. This review summarizes information on copepods symbiotic with mollusks from 1863 to 2025. Our compilation records a total of 342 symbiotic copepod species associated with more than 435 species of mollusks. This total includes some copepod species for which no specific host has been identified. For each copepod species, we provide details on its hosts, geographical distributions and the original references.

## Simple Summary

Symbiotic copepods usually live in the gills, mantle cavity and visceral mass of the marine mollusks, with hosts including polyplacophorans, bivalves, scaphopods, gastropods, and cephalopods. However, our understanding is limited regarding these copepods that are symbiotic with mollusks in the global oceans. To close this gap, we compiled a detailed list of copepods associated with mollusks found in the global oceans based on a review of the existing literature. This list includes information on species diversity, host associations, and geographical distributions. We report that 342 species of symbiotic copepods are associated with more than 435 species of mollusks, primarily bivalves and gastropods, with a few found in other molluscan classes. The most common order of the symbiotic copepods is Cyclopoida. However, many species have not been properly studied or collected, emphasizing the requirement for more research to completely understand the diversity of copepods symbiotic with mollusks in the global oceans.

## 1. Introduction

Copepods are one of the most species-rich animal groups on the earth and can be found in seawater or freshwater, where they lead planktonic, benthic, or parasitic lives [1,2]. To date, approximately 15,045 species have been described, about one-third of which are commensal or parasitic [3]. As the most abundant group of marine zooplankton, copepods are an indispensable component of the food chain. Within marine food webs, they play a crucial role: on one hand, they drive the operation of biogeochemical cycles, and on the other hand, they facilitate the smooth transfer of energy from primary producers to higher trophic levels. Due to the high sensitivity of copepod populations to the impacts of climate change and human activities, they have become highly valuable model organisms in the fields of ecology and ecotoxicology [4,5]. However, there are still significant gaps in our knowledge of these crucial organisms. Firstly, their taxonomic system remains unstable. Based on morphological characteristics, the classification system of Huys and Boxshall divides copepods into 10 orders [2]. However, the phylogenetic relationships among the entire subclass Copepoda and its various groups are still controversial [2]. For instance, Khodami et al. reclassified the order Poecilostomatoida into Cyclopoida, while some families formerly in Poecilostomatoida were assigned to the suborder Ergasilida within Cyclopoida [6]. Secondly, the research focus is heavily skewed. The majority of research on marine symbiotic copepods has focused on the parasitic copepods of fish, particularly genera *Caligus* Müller O.F., 1785 and *Lepeophtheirus* von Nordmann, 1832, commonly known as sea lice, which pose significant threats to aquaculture [7,8]. In contrast, relatively scant studies have been carried out regarding the symbiotic biology of copepods that associate with invertebrates [9]. This narrow focus substantially limits a comprehensive understanding of the diversity and ecological functions of these invertebrate-symbiotic groups.

Mollusks are important hosts for symbiotic copepods, with hosts spanning polyplacophorans, bivalves, scaphopods, gastropods, and cephalopods. It has been known for more than a century that mollusks are appropriate hosts for copepods [10]. Over 430 mollusk species serve as hosts, with the majority belonging to bivalves [11]. According to Boxshall and O’Reilly, around 280 copepod species are known to act as parasites or associates with mollusks, and an overwhelming proportion of these are marine species within the order Cyclopoida [12]. As a form of symbiosis, parasitism in molluscan copepods manifests in two primary types: ectoparasitism and endoparasitism [13]. Most of these copepods have cyclopiform or only slightly modified bodies, which applies particularly to ectoparasitic forms found on the gills and in the mantle cavity of the mollusks. In contrast, the more modified forms have elongate bodies, often with reduced segmentation and appendages, and in many cases, they inhabit the intestinal tracts of their hosts [14]. With the development of semi-intensive, intensive brackish water and marine aquaculture, the importance of parasitic copepods as disease-causing agents is becoming more and more obvious [15]. Consequently, understanding the biology of parasites is not only critical for advancing ecological knowledge, but also essential as a prerequisite for the conservation of host populations [16]. However, there are still many gaps in the taxonomic research on symbiotic copepods of mollusks. So far, the copepods of mollusks have only been summarized in specific sea areas of some countries, such as Korea and Japan, but a global review of these symbiotic copepods is currently lacking. In order to understand the species compositions and host distributions of copepod species, and enrich the data of marine biodiversity, we review the literature and summarize the copepod species of mollusks in a table.

## 2. Methods

A scoping review was conducted to synthesize existing knowledge on symbiotic copepods of mollusks in the global oceans. Databases were searched using a combination of the terms “mollus*”, “Bivalv*”, “Cephalopod*”, “Gastropod*”, “Polyplacophora*”, “Monoplacophora*”, “Aplacophora*”, “Scaphopod*”, “parasit*”, “symbio*”, “associ*” and “copepod*”. For each search result obtained, the abstract and introduction were reviewed to determine relevance. In addition to the databases, gray literature from university theses and conference presentations was searched.

The data are presented as a table compiled from the literature. The symbiotic copepods are presented in alphabetical order within the categories of order, family, genus, and species, with records of their hosts, geographical locations, and references.

## 3. Results

The symbiotic copepods with mollusks recorded in this study comprise 342 species. These species are classified into five orders, 28 families, and 86 genera, including one unidentified order and one unidentified family. Six additional copepod specimens remained unidentified at the species level (Table 1; Supplementary Materials).

Cyclopoida includes 17 families and 65 genera; Siphostomatoida includes five families and 11 genera; Harpacticoida includes four families and seven genera; and Monstrilloida includes one family and two genera. These copepods have a symbiotic relationship with most groups of mollusks, including polyplacophorans, bivalves, scaphopods, gastropods, and cephalopods. Among these hosts, bivalves constitute the most common host group, with over 205 species identified, followed by approximately 165 species of gastropods and about 65 species from other molluscan classes. Among these symbiotic copepods, Cyclopoida and Siphostomatoida are primarily associated with bivalves; Harpacticoida is found predominantly in cephalopods; and Monstrilloida occurs mainly in gastropods.

**Figure 1:**
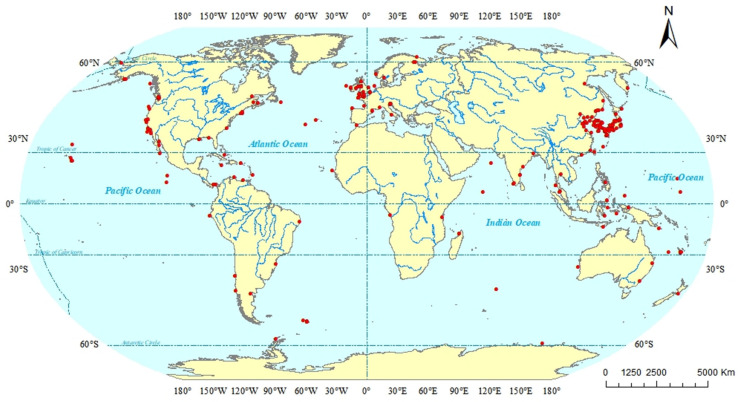
Distribution map of sampling sites. Note: Records without coordinates (data not available) not marked on the map.

**Figure 2:**
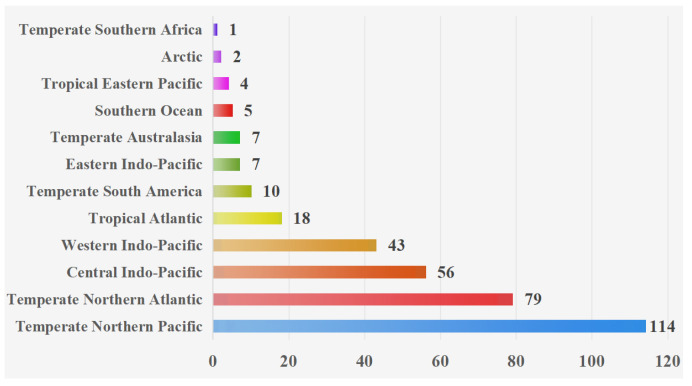
The number of symbiotic copepods of mollusks in various realms. Note: Species without clearly defined realms are not represented in the diagram.

**Figure 3:**
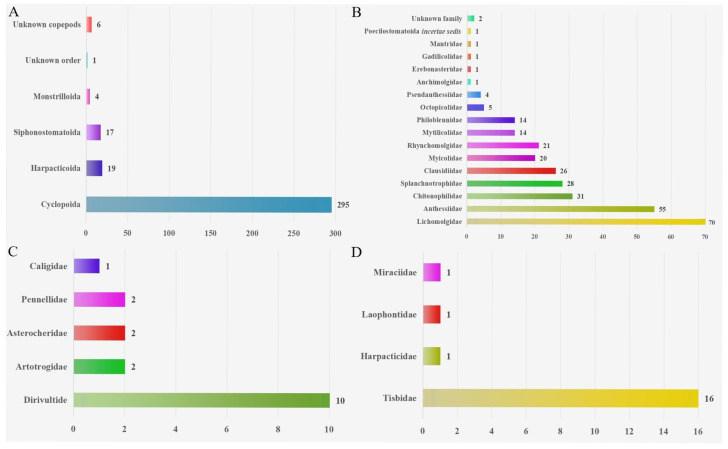
(**A**): Order-level distribution of symbiotic copepods. (**B**): Composition of families in Cyclopoida. (**C**): Composition of families in Siphonostomatoida. (**D**): Composition of families in Harpacticoida.

Based on the geographical distributions of copepods symbiotic with mollusks, a collection area map (as depicted in Figure 1) was constructed. Through this map, the specific geographical locations of the symbiotic copepods can be directly discerned. Following the classification scheme by Spalding et al., the marine ecoregions are divided into 12 realms [183,184]. Copepods symbiotic with mollusks are widely distributed across diverse ecosystems and exhibit a global geographic range (Figure 2). The majority of documented observations are concentrated in the Temperate Northern Pacific (114 species) and the Temperate Northern Atlantic (79 species), while research data from regions such as the Temperate Southern Africa (one species) and the Arctic (two species) remain relatively scarce. In this paper, the Cyclopoida is the dominant order (295 species), followed by the Harpacticoida (19 species), Siphonostomatoida (17 species), Monstrilloida (four species), and one unidentified order (one species). In addition, six specimens remained unidentified at the species level (Figure 3). Among the symbiotic copepods, the order Cyclopoida accounts for the largest proportion and the order Monstrilloida has the smallest proportion.

## 4. Discussion

Copepods are usually small and inconspicuous aquatic crustaceans, but they are extremely numerous. Due to the economic value of hosts, most studies on marine symbiotic copepods in the world have mainly focused on copepods of fish, while relatively few studies have been conducted on the copepods symbiotic with invertebrates [9].

In this review, symbiotic copepods of mollusks are found in 42 countries (as shown in Table 1). Among them, the copepods of mollusks from Japan account for the largest proportion, which may be related to its geographical location and economic condition. Japan is bordered by the Pacific Ocean to the east and the Sea of Japan to the west, which provides this country with abundant fishery resources and important sea transportation routes. Consequently, it has certain advantages in the research on copepods symbiotic with mollusks compared with other countries. Symbiotic copepods are distributed in Pacific Ocean, Atlantic Ocean, Indian Ocean and Arctic Ocean (as shown in Table 1 and Figure 1). Among them, the largest number of known symbiotic copepod species has been found in the Pacific Ocean, while the fewest have been found in the Arctic Ocean. This low diversity in the Arctic Ocean is likely related to its harsh climatic conditions. Due to the cold climate in the Arctic Ocean, scientific research work is rarely carried out.

Copepods symbiotic with mollusks are predominantly found in the Temperate Northern Pacific and the Temperate Northern Atlantic. This distribution pattern may be attributed to the complex coastlines of these regions, which feature diverse habitat types. The high habitat heterogeneity provides a wider range of ecological niches and host options for different symbiotic copepods. Moreover, these areas are home to many developed nations (e.g., Japan and the United States) and developing nations (e.g., China). These countries possess a long-standing tradition in oceanographic research, substantial funding, and well-established scientific institutions. These factors collectively contribute to the particularly rich species records in these regions. In contrast, the scarcity of research data from the Temperate Southern Africa and the Arctic may be due to limited research resources and marine science infrastructure in coastal countries of the southern temperate zone, leading to a lack of systematic marine biological surveys. The extreme environmental conditions in the Arctic, characterized by high sampling difficulty, prohibitive costs, and extensive sea ice cover for most of the year, further restrict fieldwork opportunities.

The four orders of Copepoda (Cyclopoida, Harpacticoida, Siphonostomatoida and Monstrilloida) have been reported [14,176,177]. Among the symbiotic copepods, the order Cyclopoida accounts for the largest proportion (86.09%). The remaining groups are Harpacticoida (5.63%), Siphonostomatoida (5.02%) and Monstrilloida (1.19%) (Figure 3). The reasons may be that the biological characteristics of cyclopoid copepods make them more suitable for living in molluscan hosts, and may also be that copepods of other orders have not been thoroughly discovered. In addition, compared with the more than 100,000 species of marine mollusks worldwide [185], a large number of molluscan hosts remain to be examined, which will facilitate the discovery of more unknown species. Several symbiotic copepod taxa exhibit distinct host specificity. For example, *Cholidya polypi* Farran, 1914 is currently known to be associated with cephalopods, with no records from hosts of other classes [171], thus demonstrating strict host specificity. In contrast, *Pseudomyicola spinosus* (Raffaele and Monticelli, 1885) is a symbiont associated with over 50 species of bivalves [114], exhibiting a broad host range. It is possible that the few symbionts found in some molluscan groups are due to inadequate research, thereby underestimating their symbiotic diversity. Some copepod groups that associate with only a few molluscan hosts may have evolved highly specialized attachment structures or life cycles, restricting them to utilizing specific host types.

There are also relevant reports on the impact of symbiotic copepods, particularly those that are parasitic on mollusks. Ho and Zheng described *Ostrincola koe* Tanaka, 1961 as the primary cause of mass mortalities of the cultured hard clam *Meretrix meretrix* (Linnaeus), which occurred in 1988 and 1989 in China [186]; *Pectinophilus ornatus* Nagasawa et al., 1988, a pathogen in northern Japan, parasitizes the gills of the Japanese scallop *Mizuhopecten yessoensis* (Jay), reaching infection rates of up to 100% in young scallops [136,187]; *Mytilicola intestinalis* Stuer, 1902, a copepod parasite of the gut of mussels, is endemic along European coasts and has been responsible for heavy mortalities [188]; *Mytilicola orientalis* Mori, 1935 can damage the inner wall of the intestine of *Magallana gigas* (Thunberg), leading to the death of oysters and a consequent reduction in production [189]. Currently, the marine biological diseases resulting from parasitic copepods are becoming increasingly severe and meriting greater attention. To undertake this task effectively, researchers must be well-versed in the latest developments in parasitology-related disciplines, including biology, ecology, phylogeny, and zoogeography. It is essential to promote international collaboration among experts from various countries to conduct more in-depth research on these significant symbiotic copepods and effectively prevent the diseases they cause.

## 5. Conclusions

In summary, this review offers a comprehensive survey of the species of symbiotic copepods of mollusks in the global oceans. To date, 342 species of these symbiotic copepods have been identified within more than 435 molluscan hosts. Bivalves are the most common host group. Among the symbiotic copepods, the majority (86.25%) belong to Cyclopoida, while the remaining portions consist of Harpacticoida (5.55%), Siphonostomatoida (4.97%) and Monstrilloida (1.19%). The Temperate Northern Pacific (especially the waters around Japan) contains the most known symbiotic copepod species, while the Temperate Southern Africa contains the fewest. This pattern likely reflects disparities in research effort, not actual biodiversity. The review also details the host species, geographical locations and compiles a bibliography of symbiotic copepods, thereby extending our comprehension of these organisms. It is highly probable that more species of symbiotic copepods as well as a richer diversity of species will be discovered in the future. Nevertheless, our understanding of the impacts of these symbiotic copepods on their molluscan hosts remains limited. The majority of species have been reported to do harm to the economically significant mollusks. It is imperative to highlight that further research and exploration are essential to enhance our understanding and to devise strategies for the prevention and control of the threats posed by these symbiotic associations.

## Figures and Tables

**Table 1:** Taxon of copepods symbiotic with mollusks in the global oceans.

Copepod	Hosts	Geographical Locations	References
**Order Cyclopoida**			
**Suborder Ergasilida**			
**Family Anchimolgidae**			
**Genus** ***Panjakus***			
*Panjakus platygyrae*	*Atrina vexillum* (B)	China: Hong Kong (Figure 1)	[17]
**Family Anthessiidae**			
**Genus** ***Anthessius***			
*Anthessius alatus*	*Tridacna gigas* (B)	France: New Caledonia (22.11 S, 166.25 E) (Figure 1); The Republic of the Marshall Islands: Eniwetok Atoll (Figure 1)	[18,19,20]
	*Tridacna maxima* (B)	France: New Caledonia (22.11 S, 166.25 E) (Figure 1); The Republic of the Marshall Islands: Eniwetok Atoll (Figure 1)	[18,19,20]
	*Tridacna noae* (B)	Indo-West Pacific (Data not available)	[18,20,21]
	*Tridacna squamosa* (B)	France: New Caledonia (22.20 S, 166.24 E) (Figure 1); The Republic of the Marshall Islands: Eniwetok Atoll (Figure 1); Indo-West Pacific (Data not available)	[18,19,20,21]
*Anthessius amicalis*	*Hippopus hippopus* (B)	The Republic of the Marshall Islands: Eniwetok Atoll (Figure 1)	[18,19,20]
	*Tridacna maxima* (B)	France: New Caledonia (22.18 S, 167.02 E) (Figure 1); Red Sea (Data not available)	[18,20,21]
	*Tridacna squamosa* (B)	France: New Caledonia (22.20 S, 166.24 E), (22.16 S, 166.25 E), (22.13 S, 166.23 E) (Figure 1); The Republic of the Marshall Islands: Eniwetok Atoll (Figure 1)	[18,20,21]
*Anthessius antarcticus*	*Curnon granulosa* (G)	Antarctica: Port Foster, Deception Island (62.59 S, 60.33 W) (Figure 1)	[22]
*Anthessius arcuatus*	*Pleurehdera stellata* (G)	Spain: Algeciras Bay, Cádiz (Figure 1)	[23]
*Anthessius arenicolus*	*Buccinum undatum* (G)	France: Manche (Figure 1)	[24]
	*Dosinia exoleta* (B)	France: Brittany (Figure 1); The Netherlands (Data not available); UK: North Norfolk (Figure 1)	[10,20]
*Anthessius atrinae*	*Atrina pectinata* (B)	Korea: Korea Strait (Figure 1)	[20,25]
*Anthessius brevicauda*	*Atrina vexillum* (B)	France: New Caledonia (22.16 S, 166.25 E) (Figure 1)	[19,20]
	*Pinna* sp. (B)	Indonesia: 64.70 S, 120.23 E (Figure 1)	[19,20]
*Anthessius cucullatus*	*Aplysia dactylomela* (G)	Japan: Kagoshima Prefecture (31.35 N, 130.38 E), (31.07 N, 130.12 E) (Figure 1)	[26]
	*Aplysia juliana* (G)	Japan: Kagoshima Prefecture (31.33 N, 130.33 E) (Figure 1)	[26]
	*Aplysia kurodai* (G)	Japan: Kagoshima Prefecture (31.33 N, 130.33 E), (31.14 N, 130.26 E) (Figure 1); Korea: Wolpo, Pohang (36.12 N, 129.22 E) (Figure 1)	[26,27]
*Anthessius discipedatus*	*Hippopus hippopus* (B)	Indonesia: Moluccas (Figure 1)	[20,28]
*Anthessius distensus*	*Pteria penguin* (B)	Madagascar: Nosy Bé (Figure 1)	[20,22,29]
*Anthessius dolabellae*	*Dolabella auricularia* (G)	Madagascar: Nosy Bé (Figure 1); Philippines: Bohol (9.44 N, 124.34 E) (Figure 1)	[22,27,29]
*Anthessius fitchi*	*Chaceia ovoidea* (G)	US: Carpinteria, California (Figure 1)	[20,30]
	*Zirfaea pilsbryi* (B)	US: Coos Bay, Oregon (Figure 1)	[20,30]
*Anthessius graciliunguis*	*Austromacoma constricta* (B)	Korea (Data not available)	[31]
	*Mizuhopecten yessoensis* (B)	Korea: (Sea of Japan; Yellow Sea) (Figure 1)	[25,31,32]
	*Mytilus galloprovincialis* (B)	Japan: Himeji Harbor, Hyogo Prefecture (Figure 1)	[26,31,32,33]
	*Pecten albicans* (B)	Korea (Data not available)	[31,32]
	*Scaeochlamys squamata* (B)	Korea: (Sea of Japan; Yellow Sea) (Figure 1)	[25,31,32]
	*Solecurtus divaricatus* (B)	Korea: (Sea of Japan; Yellow Sea) (Figure 1)	[25,31,32]
*Anthessius hawaiiensis*	*Pleurobranchus* sp. (G)	US: Hawaii (Figure 1)	[24,30]
*Anthessius isamusi*	*Turbo marmoratus* (G)	Japan: Kumejima Island, Ryukyu Islands (26.17 N, 126.47 E) (Figure 1)	[26,34]
*Anthessius kimjensis*	*Solen grandis* (B)	Korea: Yellow Sea (35.51 N, 126.35 E) (Figure 1)	[25,35]
*Anthessius leptostylis*	*Buccinum undatum* (G)	Japan (Data not available)	[20,34]
*Anthessius lighti*	*Aplysia californica* (G)	US: Bodega Bay, California (Figure 1)	[30,36]
*Anthessius longipedis*	*Reishia luteostoma* (G)	Japan: Sokcho (38.10 N, 128.37 E) (Figure 1)	[37]
*Anthessius minor*	*Ensis siliqua* (B)	Italy: Naples (Figure 1); Japan (Data not available); The Netherlands (Data not available)	[10,20,34,38]
*Anthessius mytilicolus*	*Perna viridis* (B)	China: (Taiwan; Hong Kong) (Figure 1); India: Ennore Estuary (Figure 1)	[20,39]
*Anthessius navanacis*	*Navanax inermis* (G)	US: Laguna Beach, California (Figure 1)	[30,36,40]
*Anthessius nortoni*	*Diodora aspera* (G)	US: San Juan Island, Washington (Figure 1)	[30]
*Anthessius nosybensis*	*Anadara antiquata* (B)	Madagascar: Nosy Bé (Figure 1)	[41]
*Anthessius obtusispina*	*Pleurobranchaea californica* (G)	US: Santa Barbara, California (Figure 1)	[22,36,42]
*Anthessius ovalipes*	*Pleurobranchus areolatus* (G)	US: Santa Barbara, California (Figure 1)	[22]
*Anthessius pectinis*	*Mizuhopecten yessoensis* (B)	Japan: Nagasaki (Figure 1)	[26]
	*Pecten albicans* (B)	Japan: Sasebo Bay, Nagasaki (Figure 1)	[31,32,43]
*Anthessius pinctadae*	*Pinctada margaritifera* (B)	France: (Southeastern Caledonia (22.18 S, 167.02 E); New Caledonia (22.20 S, 160.25 E)) (Figure 1)	[19,26]
	*Pinctada maxima* (B)	Australia: Northern Australia (Data not available)	[34]
*Anthessius pinnae*	*Pinna bicolor* (B)	China: Hong Kong (Figure 1)	[17]
	*Pinna muricata* (B)	Madagascar: Nosy Bé (Figure 1)	[29]
*Anthessius placunae*	*Placuna placenta* (B)	India: Kakinada Bay (Figure 1)	[44]
*Anthessius pleurobrancheae*	*Pleurobranchaea meckeli* (G)	Italy: (Trieste; Naples) (Figure 1); Mediterranean Sea (Data not available)	[22,24,45]
	*Pleurobranchus marginatus* (G)	Italy: (Trieste; Naples) (Figure 1); Mediterranean Sea (Data not available)	[24]
*Anthessius pleurobranchi*	*Pleurobranchaea meckeli* (G)	Italy: Trieste (Figure 1)	[46]
*Anthessius proximus*	*Dolabrifera dolabrifera* (G)	Barbados: 13.10 N, 59.38 W (Figure 1)	[22]
	*Petalifera petalifera* (G)	The Netherlands: Piscadera Bay (Data not available)	[22]
*Anthessius rarus*	*Dolabella auricularia* (G)	Philippines: Bohol (9.44 N, 124.34 E) (Figure 1)	[27]
*Anthessius saecularis*	*Tapes literatus* (B)	Indonesia: Yapen Island (Figure 1); Papua New Guinea: New Guinea (Figure 1)	[19,47]
*Anthessius sensitivus*	*Globivasum capitellum* (G)	Japan: (Data not available); West Indies (Data not available)	[20,34,48]
*Anthessius solecurti*	*Solecurtus afroccidentalis* (B)	The English Channel (Data not available)	[24,25]
*Anthessius solidus*	*Tridacna squamosa* (B)	Madagascar (Data not available); The Republic of the Marshall Islands: Eniwetok Atoll (Figure 1); Red Sea (Data not available)	[18,19,21]
*Anthessius stylocheili*	*Stylocheilus longicauda* (G)	Madagascar: Nosy Komba (Figure 1)	[22,29]
*Anthessius teissieri*	*Buccinum undatum* (G)	France: Channel coast of France (Data not available); The Netherlands (Data not available)	[10,20]
*Anthessius tuberculatus*	*Asaphis violascens* (B)	Micronesia: Kosrae (5.16 N, 162.57 E) (Figure 1)	[27]
*Anthessius varidens*	*Aplysia dactylomela* (B)	West Indies (Data not available)	[22]
	*Bursatella leachii* (G)	West Indies (Data not available)	[22]
*Anthessius* sp.	*Archidoris nivalis* (G)	UK: South Georgia Island (Figure 1)	[24]
*Anthessius* sp.	Unknown tectibranch (G)	US: California (Figure 1)	[24,36]
**Genus** ***Katanthessius***			
*Katanthessius delamarei*	*Marionia blainvillea* (G)	France: Banyuls (Figure 1)	[36,49]
*Katanthessius stocki*	*Geitodoris heathi* (G)	US: California (33.43 N, 118.23 W) (Figure 1)	[36]
**Genus** ***Neanthessius***			
*Neanthessius renicolis*	*Marmorofusus nigrirostratus* (G)	Japan: Seto (Figure 1)	[50]
	*Pleuroploca audouini* (G)	Japan: Seto (Figure 1)	[50]
**Genus** ***Panaietis***			
*Panaietis bobocephala*	*Haliotis asinina* (G)	Sea of Japan (Data not available)	[51]
*Panaietis doraconis*	*Tectus pyramis* (G)	Sea of Japan (Data not available)	[52]
*Panaietis flavellata*	*Angaria neglecta* (G)	Sea of Japan (Data not available)	[51]
*Panaietis haliotis*	*Haliotis discus* (G)	Sea of Japan (Data not available)	[53]
	*Haliotis gigantea* (G)	Sea of Japan (Data not available)	[52]
*Panaietis incamerata*	*Rochia nilotica* (G)	India: Islands of Andaman (Figure 1)	[50]
	*Turbo cornutus* (G)	Sea of Japan (Data not available)	[50]
	Unknown gastropod (G)	Papua New Guinea: Panaieti, Louisiade Archipelago (Figure 1)	[24]
*Panaietis malleolata*	*Cuspidaria obesa* (B)	Norway (Data not available)	[24]
*Panaietis satsuma*	*Tectus conus* (G)	Sea of Japan (Data not available)	[52]
	*Tectus pyramis* (G)	Sea of Japan (Data not available)	[52]
*Panaietis yamagutii*	*Turbo cornutus* (G)	Japan: (Sugari; Kiinagashima) (Figure 1); Korea: (Ullung Island; Sogwipo) (Figure 1)	[37,50]
**Family Clausidiidae**			
**Genus** ***Conchyliurus***			
*Conchyliurus bhimilensis*	*Meretrix casta* (B)	India: Quilon, Kerala (Figure 1)	[54]
*Conchyliurus bombasticus*	*Atactodea striata* (B)	India: Quilon, Kerala (Figure 1)	[55,56,57,58]
	*Meretrix meretrix* (B)	India: Puddupeta near Portonovo, Quilon, Kerala (Figure 1); Thailand: Phuket (Figure 1)	[55,56,57,58]
*Conchyliurus cardii*	*Acanthocardia echinata* (B)	UK: (Dublin Bay; Bigbury Bay) (Figure 1)	[59,60]
	*Petricolaria pholadiformis* (B)	UK: Dublin Bay (Figure 1)	[59]
	*Scrobicularia plana* (B)	UK: Dublin Bay (Figure 1)	[59]
	*Venerupis corrugata* (B)	France: Brittany (Figure 1); The Netherlands (Data not available)	[10]
*Conchyliurus dispar*	*Barnea manilensis* (B)	Korea: Yellow Sea (36.23 N, 126.32 E) (Figure 1)	[61]
*Conchyliurus inchonensis*	*Dosinia penicillata* (B)	Korea: Jakyakdo Island (Figure 1)	[25,62]
*Conchyliurus lobatus*	*Cardiocardita ajar* (B)	West Africa (Data not available)	[63]
*Conchyliurus mactrae*	*Mactra chinensis* (B)	Korea: (Sea of Japan; Yellow Sea) (Figure 1); Russia: Possiet Bay (Figure 1)	[25,64]
*Conchyliurus maximus*	*Hiatula diphos* (B)	India: Quilon, Kerala (Figure 1)	[56]
*Conchyliurus quintus*	*Anadara kagoshimensis* (B)	Korea: (Kangreung; Inchon) (Figure 1)	[64]
	*Barnea manilensis* (B)	Korea: (Sea of Japan; Yellow Sea; Korea Strait) (Figure 1)	[25]
	*Coecella chinensis* (B)	Japan: Sasebo Bay	[32]
	*Cryptomya busoensis* (B)	Korea (Data not available)	[65]
	*Cyclina sinensis* (B)	Japan: Hiroshima (Figure 1); Korea: (Inchon; Pusan; Sea of Japan; Yellow Sea; Korea Strait) (Figure 1)	[25,32,64]
	*Heteromacoma irus* (B)	Korea: (Sea of Japan; Yellow Sea; Korea Strait) (Figure 1)	[25]
	*Macoma contabulata* (B)	Japan: (Ashizaki, Mutsu City, Aomori Prefecture; Hiroura, Natori City, Miyagi Prefecture) (Figure 1)	[32]
	*Mactra chinensis* (B)	China: (Laizhou, Shandong; Qingdao, Shandong; Haiyang, Shandong; Lianyungang, Jiangsu; Nantong, Jiangsu) (Figure 1)	[66]
	*Mactra quadrangularis* (B)	China: (Qingdao, Shandong; Lianyungang, Jiangsu; Nantong, Jiangsu) (Figure 1); Japan: (Minami-Arao, Arao City, Kumamoto Prefecture; Kojiro, Shimabara City, Nagasaki Prefecture) (Figure 1); Korea: (Inchon; Sea of Japan; Yellow Sea; Korea Strait) (Figure 1)	[25,32,64,66]
	*Meretrix lusoria* (B)	Japan: Ariake Bay (Figure 1); Korea: (Puan; Sea of Japan; Yellow Sea; Korea Strait) (Figure 1)	[25,32,64]
	*Mya arenaria* (B)	Korea: (Sea of Japan; Yellow Sea; Korea Strait) (Figure 1)	[25]
	*Neotrapezium liratum* (B)	China: Lianyungang, Jiangsu (Figure 1); Japan: Rokkaku River, Ogi City, Saga Prefecture (Figure 1)	[66]
	*Nuttallia japonica* (B)	Japan: (Uno-o, Souma City, Fukushima Prefecture; Gamo, Sendai City, Miyagi Prefecture) (Figure 1); Korea (Data not available)	[32]
	*Nuttallia obscurata* (B)	Korea: (Sea of Japan; Yellow Sea; Korea Strait) (Figure 1)	[25,65]
	*Paratapes undulatus* (B)	Japan: Nagasaki (Figure 1)	[32,43,56]
	*Ruditapes philippinarum* (B)	China: (Qingdao, Shandong; Lianyungang, Jiangsu); Japan: (Sakibe, Sasebo City, Nagasaki Prefecture; Sasebo Bay, Sasebo City, Nagasaki Prefecture; Kojiro, Shimabara City, Nagasaki Prefecture; Minami-Arao, Arao City, Kumamoto Prefecture) (Figure 1); Korea: (Inchon; Puan; Mokpo; Pusan; Sea of Japan; Yellow Sea; Korea Strait) (Figure 1)	[25,32,56,64]
	*Serratina capsoides* (B)	Japan: Hiroshima (Figure 1)	[32]
	*Sinonovacula constricta* (B)	Japan: Ariake Bay (Figure 1); Korea: (Sea of Japan; Yellow Sea; Korea Strait) (Figure 1)	[25,32]
	*Solen grandis* (B)	Korea: (Sea of Japan; Yellow Sea; Korea Strait) (Figure 1)	[25]
	*Solen strictus* (B)	Korea: (Sea of Japan; Yellow Sea; Korea Strait) (Figure 1)	[25]
*Conchyliurus solenis*	*Solen marginatus* (B)	France: Brittany (Figure 1); The Netherlands (Data not available); UK: Dublin Bay (Figure 1)	[10,59,67,68,69]
*Conchyliurus torosus*	*Atactodea striata* (B)	West Africa (Data not available)	[63]
**Genus** ***Hemicyclops***			
*Hemicyclops cylindraceus*	*Jactellina adamsi* (B)	France: (Manche; Roscoff) (Figure 1); The English Channel (Data not available)	[24]
	*Loripes orbiculatus* (B)	Atlantic coast of Europe (Data not available); The English Channel (Data not available)	[70]
*Hemicyclops thysanotus*	*Hermissenda crassicornis* (G)	US: California (Figure 1)	[36]
**Genus** ***Hersiliodes***			
*Hersiliodes exiguus*	*Ruditapes philippinarum* (B)	Korea: Sokcho (Figure 1)	[71]
**Genus** ***Hyphalion***			
*Hyphalion sagamiense*	*Phreagena soyoae* (B)	Japan: Sagami Bay (Figure 1)	[32]
*Hyphalion tertium*	*Calyptogena* sp. (B)	Peru: Paita, Northern Peru (5.32 S, 81.33 E) (Figure 1)	[72]
**Genus** ***Leptinogaster***			
*Leptinogaster dentata*	*Dosinia lupinus* (B)	West Africa (Data not available)	[63]
	*Macomopsis cumana* (B)	West Africa (Data not available)	[63]
*Leptinogaster digita*	*Solen corneus* (B)	Korea (Data not available); Papua New Guinea: New Guinea (Figure 1)	[47]
	*Solen grandis* (B)	Korea: (Puan; Sea of Japan; Korea Strait; Yellow Sea) (Figure 1)	[25,73]
*Leptinogaster histrio*	*Abra alba* (B)	The Netherlands (Data not available)	[10]
	*Macoma balthica* (B)	The Netherlands (Data not available)	[10]
	*Mactra corallina* (B)	France: (Boulogne; Manche) (Figure 1); The Netherlands (Data not available); The English Channel (Data not available)	[24]
*Leptinogaster inflata*	*Lucina pensylvanica* (B)	Bahamas (Data not available)	[74]
*Leptinogaster major*	*Dosinia discus* (B)	US: Beaufort, North Carolina (Figure 1)	[75]
	*Ensis leei* (B)	US: Cotuit (Figure 1)	[76]
	*Mercenaria mercenaria* (B)	US: Beaufort, North Carolina (Figure 1)	[75,76,77]
	*Mya arenaria* (B)	Canada: Prince Edward Island (Figure 1); The Netherlands (Data not available)	[10,76]
	*Spisula solidissima* (B)	US: (Wickford; Matunuck) (Figure 1)	[76,77]
	*Tagelus plebeius* (B)	US: (Cotuit; Beaufort, North Carolina) (Figure 1)	[75,76]
*Leptinogaster minuta*	*Leukoma asperrima* (B)	Panama: Pacific coast of Panama (Figure 1)	[41]
*Leptinogaster pholadis*	*Pholas dactylus* (B)	France: Brittany (Figure 1); Italy: Naples (Figure 1); The Netherlands (Data not available)	[10,24]
*Leptinogaster pruvoti*	*Aplysia cornigera* (G)	Italy: Naples (Figure 1)	[24]
	*Aplysia sydneyensis* (G)	Italy: Naples (Figure 1)	[24]
	*Aplysia* sp. (G)	Italy: Naples (Figure 1)	[24]
*Leptinogaster scobina*	*Austromacoma nymphalis* (B)	West Africa (Data not available)	[63]
	*Iphigenia laevigata* (B)	West Africa (Data not available)	[63]
	*Macomopsis cumana* (B)	West Africa (Data not available)	[63]
	*Tagelus adansonii* (B)	West Africa (Data not available)	[63]
**Genus** ***Pholadicola***			
*Pholadicola intestinalis*	*Cyrtopleura costata* (B)	US: Galveston Bay Estuary, Texas (Data not available)	[78]
**Family Erebonasteridae**			
**Genus** ***Erebonaster***			
*Erebonaster protentipes*	*Nuculana* sp. (B)	Mexico: 27.00 N, 111.24 W (Figure 1)	[79,80]
**Family Gadilicolidae**			
**Genus** ***Gadilicola***			
*Gadilicola daviesi*	*Polyschides olivi* (S)	NE Atlantic: Rockall Trough (54.34 N, 12.19 W) (Figure 1)	[12]
	*Pulsellum lofotense* (S)	NE Atlantic: Rockall Trough (54.34 N, 12.19 W) (Figure 1)	[12]
**Family Lichomolgidae**			
**Genus** ***Epimolgus***			
*Epimolgus orientalis*	*Aplysia* sp. (G)	Australia: New South Wales (Figure 1)	[81]
	*Dolabrifera brazieri* (G)	Australia: New South Wales (Figure 1)	[82]
*Epimolgus trochi*	*Hypselodoris whitei* (G)	France: New Caledonia (Figure 1)	[24]
	*Steromphala cineraria* (G)	France: Roscoff (Figure 1)	[81]
	*Steromphala umbilicalis* (G)	France: Channel coast of France (Data not available)	[82]
	*Steromphala varia* (G)	Mediterranean Sea (Data not available)	[49,81]
**Genus** ***Gelastomolgus***			
*Gelastomolgus spondyli*	*Spondylus varius* (B)	Madagascar: Nosy Bé (Figure 1)	[81]
**Genus** ***Herrmannella***			
*Herrmannella barneae*	*Barnea candida* (B)	France: (Wimereux; Boulonnais; Manche) (Figure 1); The Netherlands (Data not available); The English Channel (Data not available)	[10,24,81]
	*Ostrea edulis* (B)	France: Baie de la Forét (Figure 1); The Netherlands (Data not available)	[10,70,81,83]
	*Pholas dactylus* (B)	France: (Longues; Brittany) (Figure 1); The Netherlands (Data not available)	[10,81]
*Herrmannella bullata*	*Chlamys hastata* (B)	US: San Juan Islands, Washington (48.31 N, 122.55 W) (Figure 1)	[81]
	*Chlamys rubida* (B)	US: San Juan Islands, Washington (48.35 N, 122.51 W) (Figure 1)	[81]
*Herrmannella caribaea*	*Chama sinuosa* (B)	Jamaica (Data not available); US: Puerto Rico (Figure 1)	[81,84]
	*Pseudochama cristella* (B)	Jamaica (Data not available)	[81,84]
*Herrmannella columbiae*	*Callithaca tenerrima* (B)	US: California (Figure 1)	[81,85]
	*Tresus capax* (B)	US: Garrison Bay, San Juan Island (Figure 1)	[81]
	*Tresus nuttallii* (B)	US: California (Figure 1)	[81,85]
*Herrmannella cubensis*	*Spondylus* sp. (B)	India: Quilon, Kerala (Figure 1)	[86]
*Herrmannella dentata*	*Cyclina sinensis* (B)	Korea (Data not available)	[87]
	*Gari kazusensis* (B)	Korea (Data not available)	[87]
	*Mya arenaria* (B)	Korea: Korea Strait (Figure 1)	[25]
	*Mya japonica* (B)	Korea (Data not available)	[87]
*Herrmannella dissidens*	*Chama sinuosa* (B)	Jamaica (Data not available); US: Puerto Rico (Figure 1)	[81,84]
	*Pseudochama cristella* (B)	Jamaica (Data not available); US: Puerto Rico (Figure 1)	[81,84]
*Herrmannella duggani*	*Ostrea edulis* (B)	Ireland: (Clew Bay; Ballynakill) (Figure 1)	[83]
*Herrmannella exigua*	*Solen strictus* (B)	Korea: Yellow Sea (Figure 1)	[25]
*Herrmannella haploceras*	*Didacna eichwaldi* (B)	France: Roscoff (Figure 1)	[81,88,89]
	*Laevicardium crassum* (B)	France: Brittany (Figure 1); The Netherlands (Data not available); UK: Port Erin, Isle of Man (Figure 1)	[10,81,88,89]
*Herrmannella hiatellai*	*Hiatella arctica* (B)	Russia: Peter the Great Bay (Figure 1)	[32]
	*Panopea japonica* (B)	Japan: Yanagawa City, Fukuoka Prefecture (Figure 1)	[25,32]
*Herrmannella hoonsooi*	*Ruditapes philippinarum* (B)	Korea: Korea Strait (Figure 1)	[25]
	*Saxidomus purpurata* (B)	Korea: (Pusan; Korea Strait) (Figure 1)	[25,90]
*Herrmannella inflatipes*	*Perna perna* (B)	Congo: Pointe Noire (Figure 1)	[63,81]
*Herrmannella kodiakensis*	*Saxidomus gigantea* (B)	US: Alaska (57.31 N, 153.58 W) (Figure 1)	[91]
*Herrmannella longicaudata*	*Mizuhopecten yessoensis* (B)	Korea: (Imwon, Kangwon-do; Korea Strait; Yellow Sea) (Figure 1)	[25,32,64,65]
	*Scaeochlamys squamata* (B)	Japan: Aomori (Figure 1); Korea: (Korea Strait; Yellow Sea) (Figure 1)	[25,32]
	*Spisula sachalinensis* (B)	Russia: Coast of the Sea of Japan (Data not available)	[25,32]
	*Swiftopecten swiftii* (B)	Korea: (Yellow Sea; Korea Strait) (Figure 1)	[25,32]
*Herrmannella longichaeta*	*Mactra chinensis* (B)	Korea: (Yellow Sea; Korea Strait) (Figure 1); Russia: Coast of the Sea of Japan (Data not available)	[25,32]
	*Spisula sachalinensis* (B)	Japan: Aomori (Figure 1); Korea: (Yellow Sea; Korea Strait) (Figure 1)	[25,32]
*Herrmannella macomae*	*Macoma contabulata* (B)	Japan: Ashizaki, Mutsu City, Aomori Prefecture (41.15 N, 141.09 E) (Figure 1)	[32]
*Herrmannella mesodesmatis*	*Mesodesma donacium* (B)	Chile: Valparaíso (Figure 1)	[81,92]
*Herrmannella panopeae*	*Clinocardium nuttallii* (B)	US: Mitchell Bay, San Juan Island, Washington (Figure 1)	[81,93]
	*Panopea generosa* (B)	US: California (Figure 1)	[81,93]
	*Tresus capax* (B)	US: Mitchell Bay, San Juan Island, Washington (Figure 1)	[81,93]
	*Tresus nuttallii* (B)	US: California (Figure 1)	[81,93]
*Herrmannella pecteni*	*Aequipecten opercularis* (B)	France: Boulogne coast of France (Figure 1); The Netherlands (Data not available)	[10,81]
	*Anomia ephippium* (B)	France: Northwestern France (Data not available)	[81]
	*Flexopecten glaber* (B)	Italy: Gulf of Trieste (Figure 1)	[24,93]
	*Karnekampia sulcata* (B)	Black Sea (Data not available)	[81]
	*Mimachlamys varia* (B)	France: (Northwestern France; Brittanny) (Figure 1); Ireland (Data not available); The Netherlands (Data not available); UK (Data not available)	[10,81]
	*Nanostrea pinnicola* (B)	Black Sea (Data not available)	[83]
	*Pecten maximus* (B)	Ireland: Kilmakilloge Harbour (Figure 1)	[83]
	*Radulopecten fibrosus* (B)	Black Sea (Data not available)	[83]
*Herrmannella perplexa*	*Saxidomus nuttalli* (B)	US: California (Figure 1)	[93]
*Herrmannella protothacae*	*Leukoma thaca* (B)	Chile: 21.15 S, 70.07 W (Figure 1)	[92]
	*Mizuhopecten yessoensis* (B)	Chile (Data not available); Korea (Data not available); Sea of Japan (Data not available)	[92]
*Herrmannella rostrata*	*Aequipecten opercularis* (B)	France: Boulogne coast of France (Data not available)	[24,81]
	*Cerastoderma edule* (B)	France: Boulogne coast of France (Data not available); The Netherlands (Data not available); UK: (Morecambe Bay; Plymouth) (Figure 1)	[10,24,81,83,93]
	*Ensis siliqua* (B)	France: (Channel coast of France; Brittany) (Figure 1); The Netherlands (Data not available)	[10,81]
	*Lutraria lutraria* (B)	France: Brittany (Figure 1); The Netherlands (Data not available)	[10]
	*Mactra corallina* (B)	France: Manche (Figure 1); The Netherlands (Data not available)	[10,24]
	*Mactra stultorum* (B)	France: Pointe aux Oies (Figure 1)	[81,93]
	*Pterocardia corallina* (B)	UK: (Firth of Forth; Lancashire) (Figure 1); The English Channel (Data not available)	[24,94]
	*Solen capensis* (B)	France: Channel coast of France (Data not available)	[81]
	*Solen marginatus* (B)	France: Brittany (Figure 1); The Netherlands (Data not available)	[10]
	*Spisula solida* (B)	France: Brittany (Figure 1); The Netherlands (Data not available)	[10]
	*Spisula subtruncata* (B)	The Netherlands (Data not available)	[10]
	*Venerupis corrugata* (B)	France: Channel coast of France (Data not available); Italy: Naples (Figure 1); The Netherlands (Data not available); UK: (Firth of Forth; Lancashire) (Figure 1)	[10,24,81,94]
*Herrmannella saxidomi*	*Leukoma staminea* (B)	US: Kodiak Island (Figure 1)	[91]
	*Saxidomus gigantea* (B)	US: Larsen Bay, Kodiak Island, Alaska (57.31 N, 153.58 W) (Figure 1)	[91]
	*Saxidomus nuttalli* (B)	US: California (Figure 1)	[81,91,93]
*Herrmannella soleni*	*Mactra quadrangularis* (B)	Japan: (Minami-Arao, Arao City, Kumamoto Prefecture; Kojiro, Shimabara City, Nagasaki Prefecture) (Figure 1); Korea: (Yellow Sea; Korea Strait) (Figure 1)	[25,32]
	*Saxidomus purpurata* (B)	China: (Rongcheng, Shandong; Haiyang, Shandong) (Figure 1)	[66]
	*Solecurtus divaricatus* (B)	Korea: (Yellow Sea; Korea Strait) (Figure 1)	[25,32]
	*Solen grandis* (B)	Korea: Puan (Figure 1)	[25,32,73]
	*Solen strictus* (B)	Korea: (Yellow Sea; Korea Strait) (Figure 1)	[25,32]
*Herrmannella tivelae*	*Tivela stultorum* (B)	US: California (Figure 1)	[81,93]
**Genus** ***Lichomolgus***			
*Lichomolgus angustus*	*Pteria penguin* (B)	Madagascar: Nosy Bé (Figure 1)	[41]
*Lichomolgus arcanus*	*Crassostrea tulipa* (B)	Senegal (Data not available)	[63,81]
	*Pitar tumens* (B)	Senegal (Data not available)	[63,81]
	*Senilia senilis* (B)	Senegal (Data not available)	[63,81]
*Lichomolgus asaphidis*	*Anachis rugosa* (G)	Madagascar: Nosy Bé (Figure 1)	[81]
	*Eastonia solanderi* (B)	Madagascar: Nosy Bé (Figure 1)	[81]
	*Solen corneus* (B)	Madagascar: Nosy Bé (Figure 1)	[81]
*Lichomolgus bidentipes*	*Arcuatula senhousia* (B)	Japan: Minami-Arao, Arao City, Kumamoto Prefecture (Figure 1)	[32]
	*Corbicula japonica* (B)	Japan: Okinohata River, Yanagawa City, Fukuoka Prefecture (Figure 1)	[32]
	*Mactra quadrangularis* (B)	Japan: Minami-Arao, Arao City, Kumamoto Prefecture (Figure 1)	[32]
	*Modiolus modulaides* (B)	Japan: Kojiro, Shimabara City, Nagasaki Prefecture (Figure 1)	[32]
	*Mytilisepta virgata* (B)	Japan: Sirahama, Wakayama Prefecture (Figure 1)	[32,64,95]
	*Mytilus galloprovincialis* (B)	Japan: Omura Bay, Nagasaki Prefecture (Figure 1); Korea (Data not available)	[32,64]
	*Ruditapes philippinarum* (B)	Japan: (Minami-Arao, Arao City, Kumamoto Prefecture; Kojiro, Shimabara City, Nagasaki Prefecture) (Figure 1)	[32]
*Lichomolgus bullatus*	*Didimacar tenebrica* (B)	Korea: Yellow Sea (Figure 1)	[25]
*Lichomolgus chamarum*	*Chama limbula* (B)	France: New Caledonia (22.13 S, 166.24 E) (Figure 1); Madagascar: Nosy Bé (Figure 1)	[19,82,96,97]
*Lichomolgus elegantulus*	*Pteria hirundo* (B)	France: Banyuls, Mediterranean coast of France (Figure 1)	[49,81]
*Lichomolgus hippopi*	*Hippopus hippopus* (B)	Indonesia: Moluccas (Figure 1)	[28]
*Lichomolgus hoi*	*Periglypta puerpera* (B)	Papua New Guinea: New Guinea (Figure 1)	[47]
*Lichomolgus ieversi*	*Flexopecten glaber* (B)	France: New Caledonia (22.13 S, 166.24 E) (Figure 1)	[19]
*Lichomolgus inflatus*	*Dosinia japonica* (B)	Korea: Southern coast of Korea (Data not available)	[25,32,98]
	*Paphia* sp. (B)	Japan: Sasebo Bay, Nagasaki Prefecture (Figure 1)	[32,43,81]
	*Pinctada fucata* (B)	Japan: Sasebo Bay, Nagasaki Prefecture (Figure 1)	[32,81,99]
	*Ruditapes philippinarum* (B)	Japan: Sasebo Bay, Nagasaki Prefecture (Figure 1)	[32,81]
	*Saxidomus purpurata* (B)	Japan: Sasebo Bay, Nagasaki Prefecture (Figure 1)	[32,81]
*Lichomolgus leptodermatus*	*Laevicardium crassum* (B)	The Netherlands (Data not available); UK: Plymouth (Figure 1)	[10,60,81]
*Lichomolgus minor*	*Pinctada* sp. (B)	Sri Lanka: Ceylon (Figure 1)	[81]
*Lichomolgus sadoensis*	*Mytilisepta virgata* (B)	Japan: Sado lsland, Niigata Prefecture (Figure 1)	[32,95]
	*Mytilus galloprovincialis* (B)	Japan: Sado Island, Niigata Prefecture (Figure 1)	[32,100]
*Lichomolgus sepicola*	*Sepia officinalis* (C)	Italy: Trieste (Figure 1)	[81]
*Lichomolgus similis*	*Cyclina sinensis* (B)	Korea: (Inchon; Yellow sea) (Figure 1)	[25,64]
	*Meretrix lusoria* (B)	Korea: Puan (Figure 1)	[25,64]
	*Meretrix meretrix* (B)	Thailand: Phuket (Figure 1); Indian Ocean (Data not available)	[58]
	*Meretrix petechialis* (B)	China: (Donggang, Liaoning; Laizhou, Shandong; Lianyungang, Jiangsu; Yancheng, Jiangsu; Nantong, Jiangsu) (Figure 1)	[66]
*Lichomolgus spondyli*	*Spondylus amanoi* (B)	Japan: Kyushu (Figure 1)	[81,101]
	*Spondylus squamosus* (B)	Japan: Wakayama Prefecture (Figure 1)	[32,101]
*Lichomolgus tridacnae*	*Tridacna gigas* (B)	The Republic of the Marshall Islands: Eniwetok Atoll (Figure 1)	[18,19,81]
*Lichomolgus uncus*	*Mytilus edulis* (B)	Australia (Data not available)	[102]
	*Perna canaliculus* (B)	New Zealand (Data not available)	[102]
*Lichomolgus* sp.	*Digitobranchus nebae* (G)	France: New Caledonia (Figure 1)	[24,81]
*Lichomolgus* sp.	*Hypselodoris whitei* (G)	France: New Caledonia (Figure 1)	[81]
**Genus** ***Modiolicola***			
*Modiolicola avdeevi*	*Gregariella difficilis* (B)	Korea: Sokcho (Figure 1)	[25,103]
*Modiolicola bifida*	*Anadara broughtonii* (B)	China: (Laizhou, Shandong; Haiyang, Shandong; Qingdao, Shandong; Lianyungang, Jiangsu) (Figure 1); Korea: (Kangreung; Chungmu; Yellow Sea) (Figure 1)	[25,64,66]
	*Anadara kagoshimensis* (B)	Korea: Kangnung (Figure 1)	[25,64]
	*Arcuatula senhousia* (B)	China: (Laizhou, Shandong; Haiyang, Shandong; Qingdao, Shandong; Lianyungang, Jiangsu) (Figure 1); Japan: Minami-Arao, Arao City, Kumamoto, Prefecture (Figure 1)	[32,66]
	*Barnea dilatata* (B)	China: (Laizhou, Shandong; Haiyang, Shandong; Qingdao, Shandong; Lianyungang, Jiangsu) (Figure 1); Korea: (Sea of Japan; Yellow Sea; Korea Strait) (Figure 1)	[25,66]
	*Barnea manilensis* (B)	Korea: (Sea of Japan; Yellow Sea; Korea Strait) (Figure 1)	[25]
	*Crenomytilus grayanus* (B)	China: (Laizhou, Shandong; Haiyang, Shandong; Qingdao, Shandong; Lianyungang, Jiangsu) (Figure 1)	[25,66]
	*Cyclina sinensis* (B)	China: Haiyang, Shandong (Figure 1); Japan: Ashizaki, Mutsu City, Aomori Prefecture (Figure 1)	[32]
	*Gregariella difficilis* (B)	Japan (Data not available)	[103]
	*Leukoma jedoensis* (B)	China: (Laizhou, Shandong; Haiyang, Shandong; Qingdao, Shandong; Lianyungang, Jiangsu) (Figure 1)	[66]
	*Mactra chinensis* (B)	Japan: Matsubara; Omura Bay; Sasebo Bay (Figure 1)	[32,81,104]
	*Mactra quadrangularis* (B)	China: Lianyungang, Jiangsu (Figure 1); Japan: Minami-Arao, Arao City, Kumamoto, Prefecture (Figure 1); Korea: (Sea of Japan; Yellow Sea; Korea Strait) (Figure 1)	[25,32]
	*Magallana gigas* (B)	Korea: (Sea of Japan; Yellow Sea; Korea Strait) (Figure 1)	[25]
	*Mytilus edulis* (B)	Korea: (Sea of Japan; Yellow Sea; Korea Strait) (Figure 1)	[25]
	*Mytilus galloprovincialis* (B)	China: (Laizhou, Shandong; Haiyang, Shandong; Qingdao, Shandong; Lianyungang, Jiangsu) (Figure 1); Japan: (Himeji Harbor, Hyogo Prefecture; Toyo, Ehime Prefecture) (Figure 1); Korea: Yongsan River (Figure 1)	[32,66,73,100]
	*Nuttallia obscurata* (B)	Korea: (Sea of Japan; Yellow Sea; Korea Strait) (Figure 1)	[25]
	*Paphia* sp. (B)	Japan: Sasebo Bay, Nagasaki Prefecture (Figure 1)	[32,43,82,102]
	*Penitella gabbii* (B)	China: (Laizhou, Shandong; Haiyang, Shandong; Qingdao, Shandong; Lianyungang, Jiangsu) (Figure 1); Korea: (Sea of Japan; Yellow Sea; Korea Strait) (Figure 1)	[25,66]
	*Ruditapes philippinarum* (B)	Japan: (Ashizaki, Mutsu City, Aomori Prefecture; Sasebo Bay, Nagasaki Prefecture; Kojiro, Shimabara City, Nagasaki Prefecture) (Figure 1); Korea: (Inchon; Puan; Chungmu; Mokpo; Sea of Japan; Yellow Sea; Korea Strait) (Figure 1)	[25,32,64,81,104,105]
*Modiolicola gracilicaudus*	*Crenomytilus grayanus* (B)	Russia: Far Eastern coast of Russia (Data not available)	[32,33]
	*Mytilus coruscus* (B)	Japan: (Sado lsland, Niigata Prefecture; Obama Bay, Fukui Prefecture; Iwami, Tottori Prefecture) (Figure 1); Korea: (Sokcho; Kangreung; Sea of Japan) (Figure 1)	[25,32,64,95,100]
	*Mytilus galloprovincialis* (B)	Japan: Sado lsland, Niigata Prefecture (Figure 1)	[32,100]
*Modiolicola gracilis*	*Mytilus californianus* (B)	US: Elkhorn Slough, Monterey Bay, California (Figure 1)	[40,81]
*Modiolicola inermis*	*Aequipecten opercularis* (B)	France: (Manche; Atlantic coast of France) (Figure 1); The Netherlands (Data not available); UK: Ecosse (Figure 1); The English Channel (Data not available)	[10,24,49,81,106]
	*Crassostrea transitoria* (B)	UK: Ecosse (Figure 1); The English Channel (Data not available)	[106]
	*Pecten maximus* (B)	France: Manche (Figure 1)	[24]
*Modiolicola insignis*	*Modiolus barbatus* (B)	France: Brittany Peninsula (Figure 1)	[81]
	*Modiolus modiolus* (B)	France: Northern France (Data not available); The Netherlands (Data not available); Norway (Data not available); Sweden: Bohuslan (Figure 1); UK (Data not available); Mediterranean Sea (Data not available); The English Channel (Data not available)	[10,24,81,107]
	*Mytilus edulis* (B)	France: Northern France (Data not available); The Netherlands (Data not available); Norway (Data not available); Sweden: Bohuslan (Figure 1); UK (Data not available); Mediterranean Sea (Data not available); The English Channel (Data not available)	[10,24,81]
	*Mytilus galloprovincialis* (B)	Italy: Naples (Figure 1); Norway (Data not available); UK (Data not available); Black Sea (Data not available); Mediterranean Sea (Data not available); The English Channel (Data not available)	[24,81]
*Modiolicola maximus*	*Crassostrea transitoria* (B)	UK: Port Erin Bay (Figure 1)	[81,108]
	*Pecten maximus* (B)	France: Concarneau (Figure 1); The Netherlands (Data not available); UK (Data not available)	[10,81]
*Modiolicola peronidius*	*Peronidia albicans* (B)	Sea of Japan (Data not available)	[109]
*Modiolicola trabalis*	*Barbatia decussata* (B)	Madagascar: Nosy Bé (Figure 1)	[41,81]
**Genus** ***Neomenicola***			
*Neomenicola gibber*	*Neomenia yamamotoi* (S)	Japan: Japanese Pacific (Data not available)	[110]
**Genus** ***Paclabius***			
*Paclabius tumidus*	*Tridacna squamosa* (B)	France: Noumea (22.13 S, 166.23 E) (Figure 1); Philippines (Data not available)	[18,19,81]
	*Tridacna* sp. (B)	Philippines: Bohol (Figure 1)	[24]
**Genus** ***Paralichomolgus***			
*Paralichomolgus orbicularis*	*Platydoris cruenta* (G)	France: Noumea, New Caledonia (Figure 1)	[81]
**Genus** ***Parapanaietis***			
*Parapanaietis tegulae*	*Tegula argyrostoma* (G)	Korea: Songsanpo (Figure 1)	[37]
	*Tegula xanthostigma* (G)	Japan: Sokcho (Figure 1)	[37]
*Parapanaietis turbo*	*Turbo stenogyrus* (G)	Japan: Tokyo (Figure 1)	[111]
**Genus** ***Paraphiloconcha***			
*Paraphiloconcha meretricis*	*Meretrix lamarckii* (B)	Japan: Oharai, Ibaraki Prefecture; Sea of Japan (Figure 1)	[25,32,81,101,112]
**Genus** ***Philoconcha***			
*Philoconcha amygdalae*	*Ruditapes philippinarum* (B)	Japan: Chiba Prefecture (Figure 1)	[32,81]
*Philoconcha paphiae*	*Dosinia japonica* (B)	Korea (Data not available)	[25,98]
	*Paphia euglypta* (B)	Japan: Inland Sea, Kyushu (Figure 1)	[32,81,101]
**Genus** ***Stellicola***			
*Stellicola alabatensis*	*Gymnodoris kouaouae* (G)	France: Noumea, New Caledonia (Figure 1)	[24,81]
*Stellicola hochbergi*	*lllex coindetii* (C)	Spain (Data not available)	[113]
*Stellicola pleurobranchi*	*Pleurobranchus* sp. (G)	Palau: Archipel Palaos (Figure 1)	[24]
**Family Myicolidae**			
**Genus** ***Exostrincola***			
*Exostrincola simplex*	*Ostrea* sp. (B)	Data not available	[114]
**Genus** ***Myicola***			
*Myicola clavator*	*Crassostrea virginica* (B)	Madagascar: Nosy Bé (Figure 1)	[115]
	*Ostrea* sp. (B)	Madagascar: Nosy Bé (Figure 1)	[116,117]
*Myicola formosanus*	*Mytilus edulis* (B)	China: Taiwan (Figure 1)	[118]
*Myicola gamoensis*	*Laternula gracilis* (B)	Japan: (Gamo, Sendai City, Miyagi Prefecture; Nanakita River (38.15 N, 141.00 E); Hiroura, Natori City, Miyagi Prefecture) (Figure 1)	[32]
*Myicola intumidus*	*Dosinia penicillata* (B)	Korea: (Jakyakdo Island; Yellow Sea) (Figure 1)	[25,62]
*Myicola metisiensis*	*Mya arenaria* (B)	Canada: Little Metis (Figure 1); The Netherlands (Data not available)	[10,24]
	*Mytilus edulis* (B)	US: Carolina (Figure 1)	[74]
*Myicola ostreae*	*Magallana gigas* (B)	Japan: (Yawata, Chiba Prefecture; Tsushima Island, Nagasaki Prefecture) (Figure 1); Korea: (Puan; Sea of Japan; Yellow Sea; Korea Strait) (Figure 1)	[25,32,43]
	*Sinonovacula constricta* (B)	Korea: (Sea of Japan; Yellow Sea; Korea Strait) (Figure 1)	[25,32]
**Genus** ***Ostrincola***			
*Ostrincola binoviger*	*Ostrea* sp. (B)	Madagascar: Nosy Bé (Figure 1)	[41,117]
*Ostrincola breviseti*	*Ostrea* sp. (B)	Madagascar: Nosy Bé (Figure 1)	[41,117]
	*Saccostrea cuccullata* (B)	Malaysia: Penang (Figure 1)	[116,117,119]
*Ostrincola falcatus*	*Anadara obesa* (B)	Mexico: Vera Cruz (Figure 1)	[120]
	*Leukoma asperrima* (B)	Panama: Chiman, Darien (Figure 1)	[116,117,120]
	*Mytella guyanensis* (B)	Mexico: Vera Cruz (Figure 1)	[116,117]
*Ostrincola gracilis*	*Crassostrea virginica* (B)	US: (Beaufort, North Carolina; Louisiana Barataria Bay) (Figure 1)	[116,117,121]
	*Geukensia demissa* (B)	US: Louisiana Barataria Bay (Figure 1)	[121]
	*Geukensia granosissima* (B)	US: Louisiana Barataria Bay (Figure 1)	[116,117]
	*Ischadium recurvum* (B)	US: Louisiana Barataria Bay (Figure 1)	[116,117]
	*Mercenaria mercenaria* (B)	US: Louisiana Barataria Bay (Figure 1)	[116,117]
	*Mya arenaria* (B)	US: Massachusetts (Figure 1)	[121]
	*Tagelus plebeius* (B)	US: Cotuit (Figure 1)	[68,116,117]
*Ostrincola humesi*	*Saccostrea cuccullata* (B)	Thailand: Chonburi Province (Figure 1)	[116,117,121,122]
*Ostrincola japonica*	*Magallana gigas* (B)	Japan: (Onagawa Port, Miyagi Prefecture; Hiroshima) (Figure 1); Korea: Puan (Figure 1)	[32,64,116,117]
	*Ostrea denselamellosa* (B)	Korea: (Yellow Sea; Korea Strait) (Figure 1)	[25,117]
	*Saccostrea echinata* (B)	Japan: Sasebo Bay, Nagasaki Prefecture (Figure 1)	[43,99,116,117]
	*Saccostrea kegaki* (B)	Japan: Sasebo Bay, Nagasaki Prefecture (Figure 1)	[32]
	*Tegillarca granosa* (B)	Korea (Data not available)	[117]
*Ostrincola koe*	*Arcopagia diaphana* (B)	Japan: Hiroshima (Figure 1)	[99,116,117]
	*Barnea dilatata* (B)	Korea: (Yellow Sea; Korea Strait) (Figure 1)	[25]
	*Barnea manilensis* (B)	Korea: (Yellow Sea; Korea Strait) (Figure 1)	[25]
	*Coecella chinensis* (B)	Japan: Sasebo Bay (Figure 1)	[32,116]
	*Cryptomya busoensis* (B)	Korea: (Yellow Sea; Korea Strait) (Figure 1)	[25,32,65,117]
	*Cyclina sinensis* (B)	Japan: Hiroshima; Korea: (Inchon; Puan) (Figure 1)	[25,64,116,117]
	*Mactra quadrangularis* (B)	China: (Laizhou, Shandong; Nantong, Jiangsu; Lianyungang, Jiangsu) (Figure 1); Japan: Minami-Arao, Arao City, Kumamoto Prefecture (Figure 1); Korea: (Inchon; Puan; Yellow Sea; Korea Strait) (Figure 1)	[25,32,64,66,116,117]
	*Meretrix lusoria* (B)	China: Chang-Hua County, Taiwan (Figure 1); Japan: Ariake Bay, Kyushu (Figure 1); Korea: (Yellow Sea; Korea Strait) (Figure 1)	[25,116,117]
	*Meretrix meretrix* (B)	China: (Laizhou, Shandong; Nantong, Jiangsu; Lianyungang, Jiangsu) (Figure 1)	[66,117]
	*Meretrix petechialis* (B)	China: (Laizhou, Shandong; Nantong, Jiangsu; Lianyungang, Jiangsu) (Figure 1)	[66]
	*Paphia* sp. (B)	Japan: Tsuyazaki, Fukutsu City, Fukuoka Prefecture (Figure 1)	[32,43,117]
	*Paratapes undulatus* (B)	Japan: Sakibe, Nagasaki Prefecture (Figure 1)	[32,43,117]
	*Petricola japonica* (B)	Japan: Sasebo Bay (Figure 1)	[32,99,117]
	*Ruditapes philippinarum* (B)	Japan: (Sasebo Bay; Nagasaki Prefecture; Minami-Arao, Arao City, Kumamoto Prefecture) (Figure 1); Korea: (Puan; Inchon; Yellow Sea; Korea Strait) (Figure 1)	[25,32,64,99,116,117]
	*Serratina capsoides* (B)	Japan: Hiroshima (Figure 1)	[32]
	*Sinonovacula constricta* (B)	Japan (Data not available); Korea: (Yellow Sea; Korea Strait) (Figure 1)	[25,117]
	*Solen grandis* (B)	Korea: (Yellow Sea; Korea Strait) (Figure 1)	[25,117]
	*Solen strictus* (B)	Korea: (Yellow Sea; Korea Strait) (Figure 1)	[25,117]
	*Umbonium moniliferum* (G)	Korea (Data not available)	[117]
*Ostrincola patagonianus*	*Brachidontes rodriguezii* (B)	Argentina: San Antonio Oeste (Figure 1)	[17,116,117]
	*Mytilus platensis* (B)	Argentina: San Antonio Oeste (Figure 1)	[17,116,117]
*Ostrincola portonoviensis*	*Atactodea striata* (B)	India: Quilon, Kerala (Figure 1)	[62,116,117]
	*Hiatula diphos* (B)	India: Puddupeta, Portonovo (Figure 1)	[116,117]
	*Marcia opima* (B)	India: Portonovo (Figure 1)	[116,117]
	*Meretrix casta* (B)	India: Adyar estuary, Portonovo (Figure 1)	[116,117]
	*Meretrix meretrix* (B)	China: Jiangsu (Data not available); India: Puddupeta, Portonovo (Figure 1); Thailand: Phuket (Figure 1)	[58,116,117,123]
*Ostrincola similis*	*Magallana gigas* (B)	China: Bu-Dai, Chiay, Taiwan (Figure 1)	[116,117]
**Genus** ***Pengna***			
*Pengna bicornuta*	*Pharella acuminata* (B)	Malaysia: Georgetown, Penang (Figure 1)	[124]
**Genus** ***Pseudomyicola***			
*Pseudomyicola spinosus*	*Anadara broughtonii* (B)	Korea: (Puan; Chungmu) (Figure 1)	[25,64]
	*Anadara kagoshimensis* (B)	Korea: (Kangnung; Puan) (Figure 1)	[25,64]
	*Anadara notabilis* (B)	Data not available	[114]
	*Anadara obesa* (B)	Data not available	[114]
	*Anomalocardia flexuosa* (B)	Data not available	[114]
	*Arca zebra* (B)	Data not available	[114]
	*Argopecten irradians irradians* (B)	US: Beaufort, North Carolina (Figure 1)	[75]
	*Argopecten ventricosus* (B)	Mexico: Baja California (Figure 1)	[125]
	*Atrina pectinata* (B)	Data not available	[114]
	*Atrina rigida* (B)	US: Beaufort, North Carolina (Figure 1)	[75]
	*Austrovenus stutchburyi* (B)	Data not available	[114]
	*Barbatia decussata* (B)	Data not available	[114]
	*Barbatia virescens* (B)	Korea: (Sea of Japan; Korea Strait) (Figure 1)	[25]
	*Brachidontes domingensis* (B)	Data not available	[114]
	*Brachidontes exustus* (B)	Data not available	[114]
	*Brachidontes modiolus* (B)	Data not available	[114]
	*Chama congregata* (B)	Data not available	[114]
	*Chama sinuosa* (B)	Data not available	[114]
	*Chione cancellata* (B)	Data not available	[114]
	*Crassostrea rhizophorae* (B)	Data not available	[114]
	*Crassostrea tulipae* (B)	Data not available	[114]
	*Crassostrea virginica* (B)	US: Beaufort, North Carolina (Figure 1)	[75]
	*Dallocardia muricata* (B)	Data not available	[114]
	*Diplodonta punctata* (B)	Data not available	[114]
	*Exolaternula liautaudi* (B)	Data not available	[114]
	*Fulvia laevigata* (B)	Data not available	[114]
	*Isognomon alatus* (B)	Data not available	[114]
	*Geukensia demissa* (B)	US: Beaufort, North Carolina (Figure 1)	[75]
	*Jasypitar albidus* (B)	Data not available	[114]
	*Lamarcka imbricata* (B)	Data not available	[114]
	*Laternula gracilis* (B)	Japan: Kanazawa Bay (Figure 1)	[32,125]
	*Leukoma jedoensis* (B)	Korea: (Sea of Japan; Korea Strait) (Figure 1)	[25]
	*Mactra quadrangularis* (B)	Korea: (Sea of Japan; Korea Strait) (Figure 1)	[25]
	*Magallana gigas* (B)	Japan (Data not available); Korea: (Mukho; Chungmu; Sea of Japan; Korea Strait) (Figure 1)	[25,64,126]
	*Modiolus kurilensis* (B)	Japan: Aomori Prefecture (Figure 1)	[32,100]
	*Megapitaria maculata* (B)	Data not available	[114]
	*Mya arenaria* (B)	Korea: (Sea of Japan; Korea Strait) (Figure 1)	[25]
	*Mytilisepta virgata* (B)	Japan: Wakayama Prefecture (Figure 1)	[32,95]
	*Mytilus edulis* (B)	China: (Dalian, Liaoning; Qingdao, Shandong) (Figure 1); Korea: (Sea of Japan; Korea Strait) (Figure 1); The Netherlands (Data not available); US: Beaufort, North Carolina (Figure 1); Mediterranean Sea (Data not available)	[10,25,66,75]
	*Mytilus galloprovincialis* (B)	France: Banyuls (Figure 1); Italy: Naples (Figure 1); Japan: (Fukushima Prefecture; Tokyo Bay) (Figure 1); Korea: (Sea of Japan; Yongsan River; Korea Strait) (Figure 1); Mediterranean Sea (Data not available)	[24,25,32,33,49,73,100,127]
	*Noetia ponderosa* (B)	US: Beaufort, North Carolina (Figure 1)	[75]
	*Nuttallia obscurata* (B)	Korea: (Sea of Japan; Korea Strait) (Figure 1)	[25]
	*Ostrea chilensis* (B)	Data not available	[114]
	*Ostrea denselamellosa* (B)	Japan: (Ashizaki, Mutsu City, Aomori Prefecture; Hutami, Hyogo Prefecture) (Figure 1)	[32,101]
	*Ostrea equestris* (B)	Data not available	[114]
	*Ostrea stentina* (B)	Data not available	[114]
	*Paphies subtriangulata* (B)	Data not available	[114]
	*Pinctada radiata* (B)	Data not available	[114]
	*Pinna carnea* (B)	Data not available	[114]
	*Pitar tumens* (B)	Data not available	[114]
	*Pseudochama radians* (B)	Data not available	[114]
	*Pteria hirundo* (B)	Data not available	[114]
	*Ruditapes philippinarum* (B)	China: (Dalian, Liaoning; Qingdao, Shandong) (Figure 1)	[66]
	*Saccostrea glomerata* (B)	Data not available	[114]
	*Senilia senilis* (B)	Data not available	[114]
	*Spondylusamericanus* (B)	Data not available	[114]
	*Tegillarca granosa* (B)	Korea: (Sea of Japan; Korea Strait) (Figure 1)	[25]
	*Tetrarca boucardi* (B)	Korea: (Sea of Japan; Korea Strait) (Figure 1)	[25]
*Pseudomyicola* sp.	*Pteria hirundo* (B)	France: Banyuls (Figure 1)	[49]
**Family Mytilicolidae**			
**Genus** ***Cerastocheres***			
*Cerastocheres trochicola*	*Tectus pyramis* (G)	France: Noumea, New Caledonia (Figure 1)	[24]
**Genus** ***Mytilicola***			
*Mytilicola brachidontis*	*Arcuatula senhousia* (B)	Japan: Shizuoka Prefecture (Figure 1)	[32,101,128]
*Mytilicola fimbriatus*	*Barbatia decussata* (B)	Madagascar: Nosy Bé (Figure 1)	[129]
*Mytilicola intestinalis*	*Cerastoderma edule* (B)	The Netherlands (Data not available); UK (Data not available)	[10]
	*Crepidula fornicata* (G)	The Netherlands (Data not available); UK (Data not available)	[10]
	*Dosinia exoleta* (B)	France: Brittanny (Figure 1); The Netherlands (Data not available)	[10]
	*Magallana gigas* (B)	Ireland (Figure 1); Wadden Sea (Data not available)	[130]
	*Mytilus edulis* (B)	The Netherlands (Data not available)	[10,24]
	*Mytilus galloprovincialis* (B)	Gulf of Trieste (Figure 1)	[24,131]
	*Spisula solida* (B)	The Netherlands (Data not available); UK (Data not available)	[10]
*Mytilicola mactrae*	*Mactra quadrangularis* (B)	Japan: Kisarazu, Chiba Prefecture (Figure 1)	[32]
*Mytilicola orientalis*	*Barnea dilatata* (B)	Korea: Yellow Sea (Figure 1)	[25,132]
	*Barnea manilensis* (B)	Korea: Yellow Sea (Figure 1)	[25]
	*Crepidula fornicata* (G)	Japan: Sea of Japan (Figure 1); Korea (Data not available); The Netherlands (Data not available); US: California (Figure 1)	[10,25,131,132]
	*Leukoma staminea* (B)	Korea: Sea of Japan (Figure 1)	[25,132]
	*Magallana gigas* (B)	Canada: Barkley Sound (Figure 1); China: (Rongcheng, Shandong; Rushan, Shandong; Qingdao, Shandong) (Figure 1); France (Data not available); Ireland (Data not available); Japan: (Hiroshima Prefecture; Inland Sea (34.19 N, 132.53 E)) (Figure 1); The Netherlands (Data not available); US: California; Mediterranean Sea (Data not available)	[32,66,132,133]
	*Mytilus californianus* (B)	Japan: Sea of Japan (Figure 1); Korea: Yellow Sea (Figure 1); US: California (Figure 1)	[25,132,134]
	*Mytilus coruscus* (B)	Japan: Inland Sea (Figure 1); Korea: Yellow Sea (Figure 1)	[25,32,66,132,133]
	*Mytilus edulis* (B)	Japan: Sea of Japan (Figure 1); Korea: Yellow Sea (Figure 1); The Netherlands (Data not available); US: California (Figure 1)	[10,25,132]
	*Mytilus galloprovincialis* (B)	China: (Dalian, Liaoning; Qingdao, Shandong) (Figure 1); Japan: Tokushima Prefecture (Figure 1)	[32,66,100,133]
	*Mytilus trossulus* (B)	Canada: Barkley Sound (Figure 1)	[135]
	*Nuttallia obscurata* (B)	Japan: Sea of Japan (Figure 1); Korea (Data not available)	[25,132]
	*Ostrea edulis* (B)	Japan: Sea of Japan (Figure 1); Korea (Data not available)	[25,131,132]
	*Ostrea lurida* (B)	Japan: Sea of Japan (Figure 1); Korea (Data not available)	[25,131,132]
	*Saxidomus gigantea* (B)	Japan (Data not available); Korea (Data not available)	[25,132]
	*Umbonium costatum* (G)	Korea: Pusan (Figure 1)	[38]
*Mytilicola porrecta*	*Cerastoderma edule* (B)	France (Data not available); Germany (Data not available); Ireland (Data not available); UK (Data not available); Adriatic Sea (Data not available); Mediterranean Sea (Data not available)	[131]
	*Crepidula fornicata* (G)	France (Data not available); Germany (Data not available); Ireland (Data not available); UK (Data not available); Adriatic Sea (Data not available); Mediterranean Sea (Data not available)	[131]
	*Geukensia granosissima* (B)	US: Barataria Bay sides of Grand Isle and Grand Terre (Figure 1)	[131]
	*Ischadium recurvum* (B)	US: Barataria Bay sides of Grand Isle and Grand Terre (Figure 1)	[131]
	*Mytilus galloprovincialis* (B)	Europe (Data not available); France (Data not available); Germany (Data not available); Ireland (Data not available); UK (Data not available); Adriatic Sea (Data not available); Mediterranean Sea (Data not available)	[131]
	*Ostrea edulis* (B)	France (Data not available); Germany (Data not available); Ireland (Data not available); UK (Data not available); Adriatic Sea (Data not available); Mediterranean Sea (Data not available)	[131]
	*Polititapes abichii* (B)	France (Data not available); Germany (Data not available); Ireland (Data not available); UK (Data not available); Adriatic Sea (Data not available); Mediterranean Sea (Data not available)	[131]
	*Steromphala cineraria* (G)	France (Data not available); Germany (Data not available); Ireland (Data not available); UK (Data not available); Adriatic Sea (Data not available); Mediterranean Sea (Data not available)	[131]
	*Steromphala varia* (G)	France (Data not available); Germany (Data not available); Ireland (Data not available); UK (Data not available); Adriatic Sea (Data not available); Mediterranean Sea (Data not available)	[131]
**Genus** ***Noetiphilus***			
*Noetiphilus elongatus*	*Noetia ponderosa* (B)	US: Beaufort, North Carolina (Figure 1)	[75]
**Genus** ***Pectenophilus***			
*Pectenophilus ornatus*	*Chlamys farreri* (B)	Japan: Southern coast of Hokkaido (Figure 1)	[32,136,137]
	*Mizuhopecten yessoensis* (B)	Japan: Southern coast of Hokkaido; Northern coast of Honshu (Figure 1)	[32,136,137]
**Genus** ***Trochicola***			
*Trochicola enterica*	*Calliostoma zizyphinum* (G)	Japan (Data not available); France: (St-Vaast-la-Hougue; Roscoff) (Figure 1); The Netherlands (Data not available)	[10,24,138]
	*Ostrea edulis* (B)	France (Data not available); US: Apalachicola Bay on the west coast of Florida (Figure 1)	[131]
	*Phorcus mutabilis* (G)	Japan (Data not available)	[138]
	*Steromphala cineraria* (G)	France: (St-Vaast-la-Hougue; Roscoff) (Figure 1); Japan (Data not available)	[24,138]
	*Steromphala varia* (G)	France: (St-Vaast-la-Hougue; Roscoff, Banyuls) (Figure 1); Japan (Data not available)	[24,49,138]
*Trochicola japonica*	*Ruditapes philippinarum* (B)	China: (Xingcheng, Liaoning; qingdao, Shandong; Nantong, Jinangsu; Lianyungang, Jinangsu) (Figure 1); Japan: (Ashizaki, Mutsu City, Aomori Prefecture; Hatsutsu-ura, Higashi-Matsushima City, Miyagi Prefecture; Lake Hamana, Shizuoka Prefecture (34.45 N, 137.35 E)) (Figure 1)	[32,138,139]
*Trochicola pectinidarum*	*Aequipecten opercularis* (B)	France: Sete (Figure 1); Japan (Data not available); The Netherlands (Data not available)	[10,138]
	*Flexopecten glaber* (B)	Japan (Data not available)	[138]
*Trochicola vermiformis*	*Gibbula magus* (G)	Japan (Data not available)	[138]
*Trochicola* sp.	*Phorcus richardi* (G)	France: Banyuls (Figure 1); Japan (Data not available)	[49,138]
**Family Octopicolidae**			
**Genus** ***Octopicola***			
*Octopicola huanghaiensis*	*Amphioctopus fangsiao* (C)	China: Shandong (Figure 1)	[139]
	*Octopus minor* (C)	China (Data not available)	[139]
*Octopicola regalis*	*Octopus cyanea* (C)	US: Beaufort, North Carolina (Figure 1)	[139]
*Octopicola stocki*	*Octopus cornutus* (C)	Madagascar: Nosy Bé (Figure 1)	[139,140]
*Octopicola superba*	*Octopus vulgaris* (C)	France: (Mediterranean coast, Roscoff; Piscadera Bay (12.07 N, 68.59 W)) (Figure 1)	[10,81,141,142]
*Octopicola* sp.	*Octopus maya* (C)	Mexico: Yucatán Peninsula (21.19 N, 33.85 W), (21.24 N, 88.02 W), (20.57 N, 90.34 W) (Figure 1)	[143]
**Family Philoblennidae**			
**Genus** ***Acanthopleuricola***			
*Acanthopleuricola sirenkoi*	*Acanthopleura gemmata* (P)	Indonesia: Karaka Island, Banda Islands, Maluku (4.30 S, 129.53 E) (Figure 1)	[144]
**Genus** ***Briarella***			
*Briarella disphaerocephala*	*Platydoris cruenta* (G)	France: New Caledonia (Figure 1)	[24,145]
	*Platydoris inframaculata* (G)	France: New Caledonia (Figure 1)	[24,145]
*Briarella doliaris*	*Ceratosoma trilobatum* (G)	Australia: 27.24 S, 153.26 E (Figure 1)	[145]
*Briarella microcephala*	*Ceratosoma trilobatum* (G)	Red Sea (Data not available)	[24,145]
	*Sclerodoris coriacea* (G)	United Republic of Tanzania: Zanzibar (Figure 1)	[145]
*Briarella risbeci*	*Ceratosoma trilobatum* (G)	Australia: 27.24 S, 153.26 E (Figure 1)	[146]
	*Hexabranchus lacer* (G)	France: New Caledonia (Figure 1)	[24,145]
	*Platydoris cruenta* (G)	France: New Caledonia (Figure 1)	[24]
*Briarella* sp.	*Asteronotus cespitosus* (G)	Philippines (Data not available)	[24]
**Genus** ***Chondrocarpus***			
*Chondrocarpus reticulosus*	Unknown pleurobranchid (G)	United Republic of Tanzania: Zanzibar (Figure 1)	[24]
*Chondrocarpus* sp.	Unknown pleurobranchid (G)	United Republic of Tanzania: Zanzibar (Figure 1)	[24]
**Genus** ***Myzotheridion***			
*Myzotheridion seguenziae*	*Carenzia carinata* (G)	Gulf of Biscay (Data not available)	[145]
**Genus** ***Nippoparasitus***			
*Nippoparasitus unoashicola*	*Patelloida saccharina* (G)	Japan: Kaneya, Futtsu City, Chiba Prefecture (Figure 1)	[145]
**Genus** ***Philoblenna***			
*Philoblenna arabici*	*Aglaja tricolorata* (G)	Japan: Seto (Figure 1)	[50]
*Philoblenna bupulda*	*Ceratostoma burnetti* (G)	Japan: Kangreung (38.40 N,128.37 E) (Figure 1)	[37,145]
	*Fusitriton oregonensis* (G)	Japan:Sokcho (Figure 1)	[37,145]
	*Ocinebrellus inornatus* (G)	Yellow sea (37.27 N, 126.37 E) (Figure 1)	[37,145]
*Philoblenna littorina*	*Littorina brevicula* (G)	Sea of Japan (Data not available)	[145]
	*Littorina mandshurica* (G)	Sea of Japan (Data not available)	[145]
	*Littorina squalida* (G)	Sea of Japan (Data not available)	[145]
*Philoblenna tumida*	*Cellana grata* (G)	Japan (Data not available); Korea (Data not available)	[52,145]
**Family Pseudanthessiidae**			
**Genus** ***Pseudanthessius***			
*Pseudanthessius dimorphus*	*Lutraria australis* (B)	Papua New Guinea: New Guinea (Figure 1)	[47]
*Pseudanthessius gracilis*	*Spisula subtruncata* (B)	France (Data not available); Italy: Trieste (Figure 1); Norway (Data not available); Sri Lanka (Data not available); Sweden (Data not available); UK: Moray Firth, Scotland (Figure 1)	[81]
*Pseudanthessius imo*	*Tectus niloticus* (G)	Japan (Data not available)	[52]
*Pseudanthessius thorellii*	*Aequipecten opercularis* (B)	UK: (Plymouth; Salcombe) (Figure 1)	[24]
**Family Rhynchomolgidae**			
**Genus** ***Critomolgus***			
*Critomolgus isoawamochi*	*Arcuatula senhousia* (B)	Japan (Data not available)	[110]
**Genus** ***Doridicola***			
*Doridicola agilis*	*Aeolidia papillosa* (G)	The Netherlands (Data not available)	[10,24,147]
	*Antiopella cristata* (G)	France: (Atlantic coast of France; Roussillon) (Figure 1); The Netherlands (Data not available); Norway (Data not available)	[10,24]
	*Aporodoris millegrana* (G)	France: Concarneau (Figure 1)	[148]
	*Armina tigrina* (G)	Senegal: 12.47 N, 17.70 W (Figure 1)	[148]
	*Cerastoderma edule* (B)	UK: Morecambe Bay, Cramond Island (Figure 1)	[81]
	*Dendrodoris limbata* (G)	Italy: Trieste (Figure 1); Adriatic Sea (Data not available)	[24,148]
	*Doris pseudoargus* (G)	France: Banyuls (Figure 1); The Netherlands (Data not available); Spain (Data not available); Sweden: Gullmarn (Figure 1)	[10,24,49,148]
	*Doris verrucosa* (G)	France: Banyuls (Figure 1); Sweden: Gullmarn (Figure 1)	[24,49,148]
	*Doto coronata* (G)	The Netherlands (Data not available)	[10]
	*Facelina auriculata* (G)	France: Atlantic coast of France (Data not available); The Netherlands (Data not available)	[10]
	*Felimare picta* (G)	Italy: Naples (Figure 1)	[81]
	*Gastropteron rubrum* (G)	France: Banyuls (Figure 1)	[49]
	*Janolus hyalinus* (G)	France: Concarneau (Figure 1)	[24,148]
	*Jorunna tomentosa* (G)	France: (Boulonnais; Concarneau) (Figure 1)	[10,148]
	*Periglypta crispata* (B)	France: Concarneau (Figure 1); UK (Data not available)	[81]
	*Proctonotus mucroniferus* (G)	France (Data not available)	[24]
	*Todarodes sagittatus* (C)	The Netherlands (Data not available); Spain: Rosas, Costa Brava (Figure 1)	[10,50]
*Doridicola antheae*	*Dendrodoris limbata* (G)	Madagascar: Nosy Bé (Figure 1)	[81]
*Doridicola audens*	*Platydoris scabra* (G)	Madagascar: Nosy Bé (Figure 1)	[81]
*Doridicola chlamydis*	*Mimachlamys varia* (B)	France: Rade de Brest (Figure 1)	[81]
*Doridicola commodus*	*Cadlina laevis* (G)	Madagascar: Nosy Bé (Figure 1)	[81]
	*Dendrodoris fumata* (G)	Madagascar: Nosy Bé (Figure 1)	[81]
	*Hexabranchus lacer* (G)	Madagascar: Nosy Bé (Figure 1)	[81,147]
	*Hexabranchus* sp. (G)	Madagascar: Nosy Bé (Figure 1)	[81]
*Doridicola gracilipes*	*Cadlina laevis* (G)	India: Rotti Island (Figure 1)	[24]
	*Gymnodoris rubromaculata* (G)	India: Rotti Island (Figure 1)	[24]
	*Hexabranchus lacer* (G)	India: Rotti Island (Figure 1)	[24]
	*Sepioteuthis lessoniana* (G)	Timor: 10.28 S, 123.24 E (Figure 1)	[81,147]
*Doridicola inflatiseta*	*Onchidium* sp. (G)	Madagascar: Nosy Bé (Figure 1)	[81]
*Doridicola larani*	*Hypselodoris* sp. (G)	Australia: Moreton Bay (Figure 1)	[149]
*Doridicola longicauda*	*Sepia officinalis* (C)	France: Nizza, Arcachon (Figure 1); Italy: Trieste (Figure 1)	[10,24,49,81]
*Doridicola parapatulus*	*Doriprismatica atromarginata* (G)	Australia (Data not available)	[25]
*Doridicola patulus*	*Phyllidia varicosa* (G)	Madagascar: Nosy Bé (Figure 1)	[81,148]
*Doridicola portincola*	*Carminodoris armata* (G)	Korea: Seogwipo Port (33.14 N, 126.33 E) (Figure 1)	[150]
*Doridicola securiger*	*Asteronotus cespitosus* (G)	Madagascar: Nosy Bé (Figure 1)	[81,147]
*Doridicola sensilis*	*Gymnodoris rubromaculata* (G)	Madagascar: Nosy Bé (Figure 1)	[81,147]
*Doridicola sepiae*	*Acanthosepion esculentum* (C)	Japan: Tanabe Bay (Figure 1); Korea (Data not available)	[147]
*Doridicola similis*	*Sepioteuthis lessoniana* (G)	Thailand: Gulf of Thailand (Figure 1)	[147]
	*Sepioteuthis* sp.(C)	Japan (Data not available)	[110]
*Doridicola venustus*	*Phyllidia varicosa* (G)	Madagascar: Nosy Bé (Figure 1)	[147]
*Doridicola virgatus*	*Pleurobranchaea japonica* (G)	Korea: Buan (35.44 N, 126.30 E) (Figure 1)	[150]
*Doridicola* sp.	*Todarodes sagittatus* (C)	Spain (Data not available)	[81]
**Genus** ***Paranthessius***			
*Paranthessius* sp.	*Mactra chinensis* (B)	Japan (Data not available)	[81]
	*Ruditapes philippinarum* (B)	Japan (Data not available)	[81]
**Family Splanchnotrophidae**			
**Genus** ***Arthurius***			
*Arthurius bunakenensis*	*Elysia pusilla* (G)	India: Gangga Island (Figure 1)	[151]
*Arthurius elysiae*	*Elysia australis* (G)	Indo-West Pacific (Data not available)	[151]
*Arthurius gibbosa*	*Elysia macnaei* (G)	Indonesia: Sulawesi (Figure 1)	[151]
**Genus** ***Ceratosomicola***			
*Ceratosomicola coia*	*Goniobranchus coi* (G)	Indonesia: Sulawesi (5.28 S, 123.43 E) (Figure 1)	[152]
*Ceratosomicola delicata*	*Goniobranchus geometricus* (G)	Indonesia: Sulawesi (5.28 S, 123.43 E) (Figure 1)	[152]
*Ceratosomicola japonica*	*Hypselodoris festiva* (G)	Japan: 34.13 N, 132.23 E (Figure 1)	[153]
*Ceratosomicola mammilata*	*Hypselodoris tryoni* (G)	Indonesia: Sulawesi (5.28 S, 123.45 E) (Figure 1)	[152]
*Ceratosomicola oki*	*Glossodoris misakinosibogae* (G)	Japan (Data not available)	[154]
*Ceratosomicola sacculata*	*Ceratosoma brevicaudatum* (G)	Australia: Houtman Abrolhos Islands (Data not available)	[152]
**Genus** ***Ismaila***			
*Ismaila aliena*	*Thecacera darwini* (G)	Chile: Bahía de Coliumo (Data not available)	[155]
*Ismaila chaihuiensis*	*Diaulula punctuolata* (G)	Chile: 39.57 S, 73.36 W (Figure 1)	[151]
*Ismaila genalis*	*Holoplocamus papposus* (G)	Chile (Data not available)	[151]
*Ismaila monstrosa*	*Antiopella fusca* (G)	US: Coos Bay, Oregon (Figure 1)	[36]
	*Doris fontainii* (G)	Chile: Temuco (Figure 1); US: St-Thomas, Antilles (Figure 1)	[24]
	*Ercolania viridis* (G)	US: California (Figure 1)	[156]
	*Melanochlamys diomedea* (G)	US: California (Figure 1)	[36]
	*Phidiana lynceus* (G)	Chile: Temuco; US: St-Thomas, Antilles (Figure 1)	[24]
*Ismaila occulta*	*Dendronotus iris* (G)	US: California (Figure 1)	[36]
*Ismaila volatilis*	*Janolus* sp. (G)	Chile (Data not available)	[151]
*Ismaila* sp.	*Doris* sp. (G)	US: California (Figure 1)	[36]
**Genus** ***Lomanoticola***			
*Splanchnotrophus brevipes*	*Coryphella verrucosa* (G)	The Netherlands (Data not available); UK: Northumberland (Figure 1); Kattegat (Data not available); North Sea (Data not available); The English Channel (Data not available)	[10,24]
	*Doto coronata* (G)	The Netherlands (Data not available); UK (Data not available)	[10,146]
	*Eubranchus tricolor* (G)	The Netherlands (Data not available); UK: Northumberland (Figure 1); Kattegat (Data not available); North Sea (Data not available); The English Channel (Data not available)	[24]
	*Trinchesia caerulea* (G)	The Netherlands (Data not available); UK: Northumberland (Figure 1); Kattegat (Data not available); North Sea (Data not available); The English Channel (Data not available)	[157]
*Lomanoticola insolens*	*Lomanotus genei* (G)	France: Marseille, Banyuls-sur-Mer (Figure 1); Ireland: Valentia (Figure 1); Italy: Naples (Figure 1); Mediterranean Sea (Data not available); The English Channel (Data not available)	[24]
	*Trinchesia caerulea* (G)	Japan: Seto (Figure 1)	[157]
*Lomanoticola nishiharai*	*Sakuraeolis enosimensis* (G)	Japan: SetoInland Sea (34.13 N, 132.23 E) (Figure 1)	[157]
	*Trinchesia caerulea* (G)	Japan: Seto (Figure 1)	[157]
**Genus** ***Majimun***			
*Majimun shirakawai*	*Roboastra gracilis* (G)	Japan: 26.26 N, 127.46 E (Figure 1)	[152]
	*Tyrannodoris luteolineata* (G)	Japan: 26.19 N, 127.44 E (Figure 1)	[152]
**Genus** ***Splanchnotrophus***			
*Splanchnotrophus angulatus*	*Aeolidia papillosa* (G)	France: (Manche; Brittanny) (Figure 1); The Netherlands (Data not available); Mediterranean Sea (Data not available); The English Channel (Data not available)	[10,24]
	*Aeolidiella glauca* (G)	France: (Manche; Brittanny) (Figure 1); The Netherlands (Data not available); Mediterranean Sea (Data not available); The English Channel (Data not available)	[10,24]
	*Cratena peregrina* (G)	The Netherlands (Data not available)	[24]
*Splanchnotrophus gracilis*	*Acanthodoris pilosa* (G)	France: (Manche; Boulonnais) (Figure 1); The Netherlands (Data not available)	[10,24,158]
	*Okenia aspersa* (G)	France: Manche (Figure 1); Ireland: Northern coasts of Ireland (Data not available)	[24]
*Splanchnotrophus helianthus*	*Thecacera pennigera* (G)	Japan: 34.19 N, 124.55 E (Figure 1)	[153]
*Splanchnotrophus imagawai*	*Trapania miltabrancha* (G)	Japan: 26.26 N, 127.54 E (Figure 1)	[153]
*Splanchnotrophus willemi*	*Ancula gibbosa* (G)	France: (Manche; Atlantic coast of France) (Figure 1); The Netherlands (Data not available)	[10,24,159]
	*Facelina auriculata* (G)	France: (Atlantic coast of France; Bay of Biscay) (Figure 1); The English Channel (Data not available)	[10,24]
*Splanchnotrophus* sp.	*Doris pseudoargus* (G)	France: Arcachon (Figure 1); The Netherlands (Data not available)	[10]
*Splanchnotrophus* sp.	*Favorinus branchialis* (G)	France: Bay of Biscay; Arcachon (Figure 1)	[24]
*Splanchnotrophus* sp.	*Favorinus branchialis* (G)	Norway: Bergen (Figure 1)	[24]
**Suborder Oithonida**			
**Family Chitonophilidae**			
Chitonophilidae gen. et sp.	*Bathyphytophilus caribaeus* (G)	NW Atlantic Ocean, from the Southeastern slope of the Grand Bahama Bank (22.24 N, 75.26 W) (Figure 1)	[160]
Chitonophilidae gen. et sp.	*Bathyphytophilus diegensis* (G)	Mexico: Baja California (32.18 N, 117.29 W) (Figure 1); NE Pacific Ocean (Data not available)	[160]
Chitonophilidae gen. et sp.	*Bathysciadium costulatum* (G)	Portugal: Azores (38.33 N, 28.19 W) (Figure 1)	[160]
Chitonophilidae gen. et sp.	*Caymanabyssia vandoverae* (G)	NE Pacific Ocean, off Oregon (44.45 N, 125.31 W) (Figure 1)	[160]
Chitonophilidae gen. et sp.	*Lepeta caeca* (G)	Sea of Japan: 43.13 N, 135.04 E (Figure 1)	[160]
Chitonophilidae gen. et sp.	*Lepetella laterocompressa* (G)	Sweden: Between Lille Sotra and Store Sotra (60.19 N, 5.80 E) (Figure 1)	[160]
Chitonophilidae gen. et sp.	*Lepetella sierrai* (G)	France: Banyuls-sur-Mer (Figure 1)	[160]
Chitonophilidae gen. et sp.	*Lepetodrilus fucensis* (G)	NE Pacific Ocean (Data not available)	[160]
Chitonophilidae gen. et sp.	*Mopalia schrencki* (P)	Russia: Vostok Bay (Figure 1); Sea of Japan (Figure 1)	[160]
Chitonophilidae gen. et sp.	*Notocrater ponderi* (G)	Australia: (New South Wales; East of Brush Island (35.33 S, 150.44 E)) (Figure 1)	[160]
**Genus** ***Chitonophilus***			
*Chitonophilus laminosus*	*Boreochiton ruber* (P)	Lesser Kurile Ridge, Kurile Islands (Figure 1)	[160]
	*Tonicella submarmorea* (P)	Japan: Sea of Japan (Figure 1); Lesser Kurile Ridge, Kurile Islands (Figure 1)	[160]
	*Tonicella zotini* (P)	Lesser Kurile Ridge, Kurile Islands (Figure 1)	[160]
**Genus** ***Cocculinika***			
*Cocculinika myzorama*	*Coccopigya hispida* (G)	New Zealand: New Zealand Island, off Castlepoint (41.09 S, 176.31 E) (Figure 1)	[160]
**Genus** ***Cookoides***			
*Cookoides cordatus*	*Stenosemus exaratus* (P)	UK: Near South Georgia Islands (53.45 S, 39.00 W) (Figure 1)	[160]
**Genus** ***Ischnochitonika***			
*Ischnochitonika aleutica*	*Belknapchiton belknapi* (P)	Bering Sea (Data not available); NW Pacific Ocean (Data not available)	[160,161]
*Ischnochitonika japonica*	*Ischnochiton hakodadensis* (P)	Russia: Sakhalin Island (Figure 1); Sea of Japan (Figure 1)	[160,161,162]
*Ischnochitonika kurochkini*	*Lepidozona multigranosa* (G)	Russia: Sea of Okhotsk (Figure 1); Sea of Japan (Figure 1)	[160,161]
	*Tripoplax albrechtii* (P)	Russia: Sea of Okhotsk (Figure 1); Sea of Japan (Figure 1)	[160,161]
	*Tripoplax kobjakovae kobjakovae* (P)	Russia: Sea of Okhotsk (Figure 1); Sea of Japan (Figure 1)	[158,159]
*Ischnochitonika lasalliana*	*Chaetopleura benaventei* (P)	Venezuela: 10.56 N, 64.12 W (Figure 1)	[160]
	*Ischnochiton striolatus* (P)	Venezuela: 10.56 N, 64.12 W (Figure 1)	[160]
	*Stenoplax boogii* (P)	Chile: 39.57 S, 73.36 W (Figure 1); Venezuela: 10.56 N, 64.12 W (Figure 1)	[160]
	*Stenoplax fallax* (P)	Venezuela: 10.56 N, 64.12 W (Figure 1)	[160]
	*Thecacera darwini* (G)	Venezuela: 10.56 N, 64.12 W (Figure 1)	[160]
	*Tonicia calbucensis* (P)	Chile: 39.57 S, 73.36 W (Figure 1)	[160]
	*Tonicia chilensis* (P)	Chile: 39.57 S, 73.36 W (Figure 1)	[160]
	*Tonicia disjuncta* (P)	Chile: 39.57 S, 73.36 W (Figure 1)	[160]
*Ischnochitonika* sp.	*Callistochiton elenensis* (P)	US: Californian Peninsula (22.57 N, 109.47 W) (Figure 1)	[160]
*Ischnochitonika* sp.	*Stenoplax marcusi* (P)	Brazil: 8.07 S, 34.48 W (Figure 1); SW Atlantic Ocean (Data not available)	[160]
**Genus** ***Lepetellicola***			
*Lepetellicola brescianii*	*Coccopigya hispida* (G)	Spain: (Galicia, Bay of Biscay; Vizcaya, Bay of Biscay (43.45 N, 8.10 W)) (Figure 1)	[160,163]
	*Lepetella sierrai* (G)	Spain: (Galicia, Bay of Biscay; Vizcaya, Bay of Biscay (43.45 N, 8.10 W)) (Figure 1)	[160,163]
**Genus** ***Leptochitonicola***			
*Leptochitonicola attenuata*	*Leptochiton rugatus* (P)	Russia: Bering Island, Commander Islands (Figure 1)	[160]
*Leptochitonicola hanleyellai*	*Hanleyella asiatica* (P)	Russia: Bering Island, Commander Islands (Figure 1)	[160]
*Leptochitonicola intermedia*	*Leptochiton* sp. (P)	Russia: 53.26 N, 160.21 E (Figure 1)	[160]
*Leptochitonicola latus*	*Leptochiton assimilis* (P)	Lesser Kurile Ridge (Figure 1)	[160]
*Leptochitonicola sphaerica*	*Leptochiton rugatus* (P)	Russia: Posyet Bay (42.30 N, 130.55 E) (Figure 1); Sea of Japan (Figure 1)	[160]
*Leptochitonicola* sp.	*Belknapchiton alveolus* (P)	Canada: Newfoundland Bank (46.40 N, 50.00 W) (Figure 1)	[160]
*Leptochitonicola* sp.	*Hanleyella oldroydi* (P)	US: California (32.59 N, 119.32 W) (Figure 1)	[160]
**Genus** ***Leptochitonoides***			
*Leptochitonoides vitiasi*	*Belknapchiton belknapi* (P)	US: Alaska (55.23 N, 134.46 W) (Figure 1)	[160,161]
**Genus** ***Nucellicola***			
*Nucellicola holmanae*	*Nucella lapillus* (G)	UK: Robin Hood’s Bay, North Yorkshire, England, North Sea (Figure 1); NE Atlantic Ocean (Data not available)	[160,164]
*Nucellicola* sp.	*Buccinum undatum* (G)	Russia: Kandalaksha Bay of the White Sea (66.17 N, 33.39 E); (66.33 N,33.06 E); (69.06 N, 36.04 E) (Figure 1)	[165]
	*Neptunea despecta* (G)	Russia: Kandalaksha Bay of the White Sea (66.17 N, 33.39 E); (66.33 N,33.06 E) (Figure 1)	[165]
**Genus** ***Tesonesma***			
*Tesonesma reniformis*	*Stenosemus albus* (P)	Bering Strait (66.02 N, 169.29 W) (Figure 1); Russia: (Shantar Island (55.33 N, 136.23 E); Strait of Tartar (47.18 N, 139.01 E)) (Figure 1); Sea of Japan (Figure 1); Sea of Okhotsk to the Bering Sea (Data not available)	[160,163]
**Family Mantridae**			
**Genus** ***Chamicola***			
*Chamicola nagasawai*	*Pseudochama retroversa* (B)	Japan (Data not available)	[166]
**Genus** ***Teredoika***			
*Teredoika aspectabilis*	*Clinopegma magnum unicum* (G)	Italy: Naples (Figure 1)	[167]
**Suborder Poecilostomatoida**			
**Family Poecilostomatoida** ***incertae sedis***			
**Genus** ***Endocheres***			
*Endocheres obscurus*	*Calliostoma zizyphinum* (G)	The Netherlands (Data not available)	[10]
**Unknown suborder**			
**Family Cyclopoida** ***incertae sedis***			
**Genus** ***Ameristocheres***			
*Ameristocheres inermis*	*Aglaja tricolorata* (G)	Italy: Naples (Figure 1)	[24]
**Order Harpacticoida**			
**Family** Harpacticidae			
**Genus Harpacticus**			
*Harpacticus* sp.	*Acanthopleura granulata* (P)	West Indian (Data not available)	[168]
	*Chiton tubereulatus* (P)	West Indian (Data not available)	[168]
**Family Laophontidae**			
**Genus** ***Heterolaophonte***			
*Heterolaophonte lalanai*	*Acanthopleura granulata* (P)	West Indian (Data not available)	[168]
	*Chiton tuberculatus* (P)	West Indian (Data not available)	[168]
**Family Miraciidae**			
**Genus** ***Amphiascus***			
*Amphiascus waihonu*	*Choristella marshalli* (G)	New Zealand: 44 55 S, 174.04 E (Figure 1)	[168]
**Family Tisbidae**			
**Genus** ***Seutellidium***			
*Scutellidium patellarum*	*Cymbula granatina* (G)	South Africa (Data not available)	[168]
	*Cymbula miniata* (G)	South Africa (Data not available)	[168]
	*Cymbula oculus* (G)	South Africa (Data not available)	[168]
	*Scutellastra argemvillei* (G)	South Africa (Data not available)	[168]
	*Scutellastra barbara* (G)	South Africa (Data not available)	[168]
	*Scutellastra cochlear* (G)	South Africa (Data not available)	[168]
	*Scutellastra longicosta* (G)	South Africa (Data not available)	[168]
	*Scutellastra tabularis* (G)	South Africa (Data not available)	[168]
**Subfamily Cholidyinae**			
**Genus** ***Amplipedicola***			
*Amplipedicola pectinatus*	*Enteroctopus dofleini* (C)	Bering Sea (Data not available)	[169]
	*Octopus vulgaris* (C)	Japan (Data not available)	[168]
**Genus** ***Avdeevia***			
*Avdeevia antarctica*	*Megaleledone setebos* (C)	UK: South Georgia (Figure 1)	[168]
**Genus** ***Brescianiana***			
*Brescianiana rotundata*	*Graneledone boreopacifica* (C)	Pacific Ocean (Data not available)	[170]
**Genus** ***Cholidya***			
*Cholidya polypi*	*Bathypolypus areticus* (C)	Ireland: Southwest coast of Ireland (Data not available)	[24,170,171]
	*Bathypolypus ergasticus* (C)	Ireland: Southwest coast of Ireland (Data not available)	[168]
	*Graneledone boreopacifica* (C)	NE Pacific (Data not available)	[168]
	*Graneledone* sp. (C)	NE Pacific (Data not available)	[168]
	*Graneledone* sp. (C)	US: California, Monterey Bay (Figure 1)	[168]
	*Tetracheledone spinicirrhus* (C)	US: North Carolina (Figure 1)	[168]
	Unknown octopodid (C)	Ireland: Southwest coast of Ireland (Data not available)	[168]
**Genus** ***Cholidyella***			
*Cholidyella breviseta*	*Opisthoteuthis californiana* (C)	Pacific coast of Honshu (Data not available)	[170]
*Cholidyella incisa*	*Graneledone boreopacifica* (C)	Pacific coast of Honshu (Data not available)	[170]
*Cholidyella intermedia*	Unidentified cirrotheutid cephalopod (C)	Faroe-Shetlands Channel (Data not available)	[170,171]
*Cholidyella nesisi*	*Muusoctopus fuscus* (C)	Pacific coast of Honshu (Data not available)	[170]
	*Muusoctopus profundorum* (C)	Pacific coast of Honshu (Data not available)	[170]
**Genus** ***Genesis***			
*Genesis vulcanoctopusi*	*Vulcanoctopus hydrothermalis* (C)	East Pacific: 12.48 N, 103.56 W (Figure 1)	[172]
**Genus** ***Octopinella***			
*Octopinella tenacis*	*Muusoctopus hokkaidensis* (C)	Kurile Region (Data not available)	[170]
	*Muusoctopus profundorum* (C)	North Kurile Region (Data not available)	[170]
	*Octopus longispadiceus* (C)	Kurile Region (Data not available)	[170]
	*Octopus* sp. (C)	Kurile Region (Data not available)	[170]
	*Sasakiopus salebrosus* (C)	Kurile Region (Data not available)	[170]
**Genus** ***Tripartisoma***			
*Tripartisoma ovalis*	*Pareledone charcoti* (C)	Ross Sea (Data not available)	[170,173]
	*Pareledone harrissoni* (C)	Ross Sea (Data not available)	[170,173]
	*Pareledone turqueti* (C)	Ross Sea (Data not available)	[170]
*Tripartisoma trapezoidalis*	*Pareledone harrissoni* (C)	Ross Sea (Data not available)	[170]
**Genus** ***Yunona***			
*Yunona marginata*	*Pareledone charcoti* (C)	Ross Sea (Data not available)	[170]
	*Pareledone harrissoni* (C)	Ross Sea (Data not available)	[170]
**Subfamily Tisbinae**			
**Genus** ***Tisbe***			
*Tisbe celata*	*Mytilus edulis* (B)	Argentina (Data not available); Canada: New Brunswick (Figure 1); The Netherlands (Data not available)	[10]
*Tisbe* sp.	*Mytilus edulis* (B)	Argentina (Data not available)	[168]
**Order Monstrilloida**			
**Family Monstrillidae**			
**Genus** ***Caromiobenella***			
*Caromiobenella helgolandica*	*Brachystomia scalaris* (G)	France: Wimereux (Figure 1); The Netherlands (Data not available)	[10,24,174]
*Caromiobenella serricornis*	*Brachystomia scalaris* (G)	North Atlantic Ocean (Data not available)	[175]
**Genus** ***Monstrilla***			
*Monstrilla* sp.	*Mytilus galloprovincialis* (B)	Japan (Data not available)	[176]
*Monstrilla* sp.	*Perna perna* (B)	Brazil: Penha, Santa Catarina State (Figure 1); China: Hong Kong (Figure 1)	[177]
**Order Siphonostomatoida**			
**Family Artotrogidae**			
**Genus** ***Artotrogus***			
*Artotrogus orbicularis*	*Doris* sp. (G)	Sri Lanka: Ceylon (Figure 1); UK (Data not available); Kara Sea (Data not available)	[24,178]
	*Platydoris cruenta* (G)	France: Noumea, New Caledonia (Figure 1)	[24]
**Genus** ***Neobradypontius***			
*Neobradypontius australis*	*Archidoris nivalis* (G)	UK: South Georgia Island (Figure 1)	[24]
**Family Asterocheridae**			
**Genus** ***Obesiella***			
*Obesiella lyonsiellae*	*Policordia papyracea* (B)	Southern Indian Ocean (Data not available)	[24,179]
**Genus** ***Scottocheres***			
*Scottocheres elongatus*	*Aequipecten opercularis* (B)	France: (Manche; Plymouth) (Figure 1); Italy: Naples (Figure 1); Norway (Data not available); Sri Lanka: Ceylon (Figure 1); UK: Ecosse (Figure 1)	[24]
**Family Caligidae**			
**Genus** ***Anchicaligus***			
*Anchicaligus nautili*	*Nautilus macromphalus* (C)	Papua New Guinea: New Britain (Figure 1)	[24]
	*Nautilus pompilius* (C)	Palau (Data not available); Philippines (Data not available)	[24,95]
**Family Dirivultidae**			
**Genus** ***Aphotopontius***			
*Aphotopontius acanthinus*	*Lepetodrilus elevatus* (G)	Eastern Pacific: 9.83 N, 104.28 W (Figure 1)	[180]
*Aphotopontius arcuatus*	Unknown bivalve (B)	Northeastern Pacifc (Data not available)	[180]
*Aphotopontius atlanteus*	Unknown bivalve (B)	Northeastern Pacifc (Data not available)	[180]
*Aphotopontius flexispinus*	Unknown bivalve (B)	Northeastern Pacifc (Data not available)	[180]
*Aphotopontius probolus*	Unknown bivalve (B)	Northeastern Pacifc (Data not available)	[180]
**Genus** ***Ceuthoecetes***			
*Ceuthoecetes acanthothrix*	Unknown bivalve (B)	Eastern Pacific (Data not available)	[180]
*Ceuthoecetes aliger*	Unknown bivalve (B)	Eastern Pacific (Data not available)	[180]
*Ceuthoecetes cristatus*	Unknown bivalve (B)	Eastern Pacific (Data not available)	[180]
**Genus** ***Exrima***			
*Exrima singula*	Unknown bivalve (B)	Eastern Pacific (Data not available)	[180]
**Genus** ***Nilva***			
*Nilva torifera*	Unknown bivalve (B)	Eastern Pacific (Data not available)	[180]
**Family Pennellidae**			
**Genus** ***Cardiodectes***			
*Cardiodectes bellottii*	*Carinaria japonica* (G)	US: California (Figure 1)	[181]
	*Cavolinia tridentata* (G)	US: California (Figure 1)	[181]
	*Clio cuspidata* (G)	US: California (Figure 1)	[181]
	*Clio pyramidata* (G)	US: California (Figure 1)	[181]
	*Clio recurva* (G)	US: California (Figure 1)	[181]
	*Cuvierina columnella* (G)	US: California (Figure 1)	[181]
	*Diacria trispinosa* (G)	US: California (Figure 1)	[181]
	*Janthina umbilicata* (G)	US: California (Figure 1)	[181]
**Genus** ***Pennella***			
*Pennella filosa*	*Eledone moschata* (C)	The Netherlands (Data not available); Adriatic Sea (Data not available)	[10]
	*Loligo vulgaris* (C)	The Netherlands (Data not available); Adriatic Sea (Data not available)	[10]
	*Sepia officinalis* (C)	Italy: Trieste (Figure 1)	[24]
	*Todaropsis eblanae* (C)	France: Rosas (Figure 1); Adriatic Sea (Data not available)	[49]
Unknown order			
Unknown family			
**Genus** ***Teredicola***			
*Teredicola typica*	*Lyrodus affinis* (B)	US: (Kahului, Maui; Honolulu, Hawaii; Hilo Harbor, Hawaii) (Figure 1)	[182]
Unknown copepod	*Diversidoris flava* (G)	France: New Caledonia (Figure 1)	[81]
	*Doris immonda* (G)	France: New Caledonia (Figure 1)	[81]
Unknown copepod	*Frenamya elongata* (B)	Indian Ocean (Data not available)	[24]
Unknown copepod	*Laternula anatina* (B)	Indian Ocean (Data not available)	[24]
Unknown copepod	*Ostrea edulis* (B)	The English Channel (Data not available)	[24]
Unknown copepod	*Pinna* sp. (B)	Indian Ocean (Data not available)	[24]
Unknown copepod	*Zirfaea crispata* (B)	The English Channel (Data not available)	[24]

Note: B: Bivalvia; C: Cephalopoda; G: Gastropoda; P: Polyplacophora; S: Scaphopoda.

## Data Availability

The data presented in this study are contained within the article.

## Supplementary Materials

The following supporting information can be downloaded at: https://www.mdpi.com/article/10.3390/ani16020212/s1, Description of the dataset with specific information relative to copepod symbiotic with mollusks, order, suborder, family, subfamily, genus, hosts, site of infection, geographical locations [dates] and references.
